# Using Machine Learning Methods to Predict Early Treatment Outcomes for Multidrug-Resistant or Rifampicin-Resistant Tuberculosis to Enhance Patient Cure Rates: Development and Validation of Multiple Models

**DOI:** 10.2196/69998

**Published:** 2025-09-22

**Authors:** Fuzhen Zhang, Zilong Yang, Xiaonan Geng, Yu Dong, Shanshan Li, Cong Yao, Yuanyuan Shang, Weicong Ren, Ruichao Liu, Haobin Kuang, Liang Li, Yu Pang

**Affiliations:** 1 Department of Bacteriology and Immunology, Beijing Chest Hospital, Capital Medical University/Beijing Tuberculosis & Thoracic Tumor Research Institute Beijing China; 2 Department of Epidemiology, School of Public Health, Cheeloo College of Medicine, Shandong University Jinan China; 3 School of Public Health (Shenzhen), Shenzhen Key Laboratory of Pathogenic Microbes and Biosafety, Shenzhen Campus of Sun Yat-sen University, Sun Yat-sen University Shenzhen China; 4 Department of Tuberculosis, Guangzhou Chest Hospital/ Guangzhou Key Laboratory of Tuberculosis Research/ State Key Laboratory of Respiratory Disease Guangzhou China

**Keywords:** multidrug-resistant or rifampicin-resistant tuberculosis, therapeutic efficacy, culture conversion, machine learning, prediction, artificial intelligence

## Abstract

**Background:**

Early prediction of treatment outcomes for patients with multidrug-resistant or rifampicin-resistant tuberculosis (MDR/RR-TB) undergoing extended therapy is crucial for enhancing clinical prognoses and preventing the transmission of this deadly disease. However, the absence of validated predictive models remains a significant challenge.

**Objective:**

This study compared a conventional logistic regression model with machine learning (ML) models using demographic and clinical data to predict outcomes at 2 and 6 months of treatment for MDR/RR-TB. The goal was to advance model applications, refine control strategies, and boost MDR/RR-TB cure rates.

**Methods:**

This retrospective study encompassed an internal cohort of 744 patients with MDR/RR-TB examined between January 2017 and June 2023, as well as an external cohort comprising 137 patients with MDR/RR-TB examined between March 2021 and June 2022. Data on culture conversion were collected at 2 and 6 months, and culture conversion was tracked in the external cohort at the same time points. The internal cohort was assigned as the training set, whereas the external cohort was used as the validation set. Logistic regression and 7 ML models were developed to predict the culture conversion of patients with MDR/RR-TB at 2 and 6 months of treatment. Model performance was evaluated using the area under the curve, accuracy, sensitivity, and specificity.

**Results:**

In the internal cohort, culture conversion rates for MDR/RR-TB were 81.9% (485/592) at 2 months and 87.1% (406/466) at 6 months. The odds ratio for treatment success was 8.55 (95% CI 3.31-22.08) at 2 months and 20.33 (95% CI 6.90-59.86) at 6 months after conversion, with sensitivities of 86.5% and 92.2% and specificities of 57.1% and 63.2%, respectively. The artificial neural network model was the best for culture conversion at both 2 and 6 months of treatment, with areas under the curve of 0.82 (95% CI 0.77-0.86) and 0.90 (95% CI 0.86-0.93), respectively. The accuracy, sensitivity, and specificity of the model were 0.74, 0.74, and 0.75 at 2 months of treatment and 0.80, 0.79, and 0.87 at 6 months of treatment, respectively.

**Conclusions:**

The ML models based on 2- and 6-month culture conversion could accurately predict treatment outcomes for patients with MDR/RR-TB. ML models, particularly the artificial neural network model, outperformed the logistic regression model in both stability and generalizability and offer a rapid and effective tool for evaluating therapeutic efficacy in the early stages of MDR/RR-TB treatment.

## Introduction

### Background

Multidrug-resistant or rifampicin-resistant tuberculosis (MDR/RR-TB; resistance to rifampicin and isoniazid or rifampicin alone) poses a significant challenge to tuberculosis (TB) control worldwide, particularly in limited-income countries [1,2]. As of 2023, the World Health Organization (WHO) estimated that 3.2% of new TB cases and 16% of retreatment cases worldwide were MDR/RR-TB [1]. Despite improvements in treatment options, including new drugs such as linezolid (LZD), bedaquiline (BDQ), and delamanid (DLM), the current cure rate for MDR/RR-TB remains at approximately 60% [3]. MDR/RR-TB treatment is more expensive, lengthier, and associated with more side effects compared to drug-susceptible TB [4]. In addition, prolonged treatment can lead to extensively drug-resistant TB, which has an 80% mortality rate [5].

Sputum culture is the gold standard for assessing TB treatment efficacy, with culture conversion indicating a favorable outcome. However, sputum cultures are susceptible to contamination and lengthy incubation periods [6]. Due to the limitations of traditional assessment methods, innovative evaluation strategies have emerged, incorporating novel biomarkers, omics approaches, and therapeutic efficacy models [7,8]. However, these advanced biomarkers and omics-based analyses often rely on laboratory testing for efficacy assessment, which can be time-consuming and costly [7]. Furthermore, although some novel biomarkers have been used to evaluate treatment outcomes in TB, their application remains at an early stage and requires further validation [9]. Consequently, it is feasible to develop efficacy evaluation models based on demographic and clinical parameters such as age, gender, and treatment regimens to predict TB treatment outcomes. Currently, some studies use patient demographic and clinical features to construct predictive models for prognosis [10-12]. However, most of these models focus on predicting the ultimate outcomes of MDR/RR-TB, with a notable gap in early efficacy prediction models specifically for MDR/RR-TB. Therefore, the use of predictive models to forecast early treatment efficacy in patients with MDR/RR-TB not only advances the development of this field but also aids in monitoring therapeutic outcomes. There is evidence suggesting that culture conversion at 2 and 6 months after treatment initiation serves as an early predictor of successful outcomes in patients with MDR/RR-TB [13], yet research in this area is still limited.

### Objectives

Machine learning (ML) algorithms based on artificial intelligence are increasingly applied in medical and public health research, showing significant potential in disease prevention, diagnosis, and treatment [14-16]. The conventional logistic regression model is widely used as a benchmark against more complex ML algorithms to assess whether the latter offer any meaningful improvement in predictive performance [17]. This study compared conventional logistic regression with several ML models using demographic and clinical data to predict outcomes at 2 and 6 months of treatment for MDR/RR-TB. The goal was to advance model applications, refine control strategies, and boost MDR/RR-TB cure rates.

## Methods

### Study Design

We conducted a retrospective cohort study collecting data from patients with MDR/RR-TB diagnosed through drug susceptibility testing or molecular diagnostic testing and who had completed 2 or 6 months of treatment at Beijing Chest Hospital, Capital Medical University, and Guangzhou Chest Hospital in China. These patients were divided into a culture conversion group and no–culture conversion group. Predictive models for assessing the efficacy after 2 or 6 months of treatment were then constructed. The inclusion and exclusion criteria for the study are presented in Textbox 1.

**Inclusion and exclusion criteria.**

**Inclusion criteria**

- Patients with a definitive laboratory diagnosis of multidrug-resistant or rifampicin-resistant tuberculosis (MDR/RR-TB; drug susceptibility testing or molecular diagnostic testing)
- Patients aged ≥16 years
- Patients who had not yet started MDR/RR treatment or had started treatment but required further adjustment due to poor efficacy
- Patients with positive sputum smear or culture at baseline

**Exclusion criteria**

- Patients with multidrug-resistant or rifampicin-resistant pulmonary TB without a definitive laboratory diagnosis
- Patients with isolated extrapulmonary TB
- Patients who had not received TB treatment
- Patients who died
- Patients with ≥80% of missing data

From January 3, 2017, to June 29, 2023, data from patients with MDR/RR-TB were collected at Beijing Chest Hospital, Capital Medical University, to form an internal training cohort. Patients were included for predicting treatment outcomes at both 2 and 6 months. Subsequently, an external validation cohort was established by collecting data from patients with MDR/RR-TB at Guangzhou Chest Hospital between March 1, 2021, and June 30, 2022. All patients with MDR/RR-TB included in this study were treated according to the WHO guidelines for MDR/RR-TB treatment regimens.

### Data Collection and Processing

Data were collected using a Microsoft Excel form from the electronic patient management system of the hospital, which included demographic information, clinical data, and the outcomes of sputum culture conversion after 2 months of treatment. We gathered general demographic characteristics, treatment-related factors, comorbidities, computed tomography imaging features, immunological indicators, results of sputum smears and cultures, and treatment outcomes for each patient. Treatment success was defined as the achievement of 2 consecutive negative sputum cultures obtained at least 30 days apart following the completion of therapy in patients with MDR/RR-TB. Conversely, treatment failure was characterized by the failure to meet these criteria. After standardizing the data, patients with ≥80% of missing data were excluded. For ≤5% of individual missing data, numerical variables were filled in using the mean, and categorical variables were filled in using the mode, ensuring the clarity, logic, and completeness of the data.

### Model Construction

Univariate logistic regression analysis was conducted on the internal training cohort treated for 2 and 6 months to identify factors affecting the sputum culture conversion outcomes in patients with MDR/RR-TB (*P*<.05). Multicollinearity and Spearman correlation analyses were conducted on these factors. A Spearman correlation coefficient of <0.4 was regarded as indicating a weak correlation. All variables with weak correlations were included in the model, whereas for those with strong correlations, only 1 variable with a greater impact on treatment outcomes was selected for model construction. The internal cohort treated for 2 and 6 months were used as the training sets to build the models, and the external cohort served as the validation sets to evaluate the models. Logistic regression, random forest (RF), support vector machine (SVM), gradient boosting decision tree (GBDT), elastic net (EN), and artificial neural network (ANN) models were developed using the selected variables. In addition, stacking and voting ensemble methods were applied to the single RF, SVM, GBDT, EN, and ANN models for ensemble learning. The predictive performance of the different models was compared.

### Statistical Analysis

Baseline characteristics and univariate logistic regression analysis of the internal cohort were conducted using SPSS (version 27; IBM Corp). For count variables, frequency counts and percentages were used, and for quantitative variables, the mean and SD or median and IQR were used based on their normal distribution. To measure differences between groups, count variables were assessed using the chi-square test or Fisher exact test, whereas quantitative variables were evaluated using 2-tailed *t* tests or Mann-Whitney *U* tests based on their distribution. The odds ratio (OR) and its 95% CI were used to assess the strength of the association between influencing factors and early treatment outcomes in patients with MDR/RR-TB. Tolerance and variance inflation factor (VIF) tests were conducted to check for multicollinearity among selected variables; a tolerance of <0.1 or a VIF of >10 indicated the presence of multicollinearity. Spearman correlation coefficient matrix analysis was conducted using the *corrplot* package in RStudio (version 4.4.1; Posit PBC).

Prediction models were constructed using Python’s scikit-learn (Google Summer of Code project) ML library using the synthetic minority oversampling technique to balance the training set and enhance predictive performance. The hyperparameters were optimized using 5-fold cross-validation combined with grid search. The optimal hyperparameters and models for the RF, SVM, GBDT, EN, and ANN models were determined. The best parameter settings for each of the 5 models are shown in Table S1 in Multimedia Appendix 1. Receiver operating characteristic curves were plotted, and 95% CIs were calculated to evaluate the predictive performance of the models through the area under the curve (AUC). In addition, the accuracy, sensitivity, specificity, and *F*_1_-scores of each model were compared, and the importance scores of variables in the explainable models were assessed. A 2-sided significance level was set, with *P*<.05.

### Ethical Considerations

This study received ethics approval from the ethics committee of Beijing Chest Hospital, Capital Medical University, under approval YJS-2022-01 in accordance with the 2013 Declaration of Helsinki. Due to the retrospective nature of the research and the use of anonymized data, the ethics committee waived the requirement for patient informed consent.

## Results

### Patients and Follow-Up

**Figure 1:**
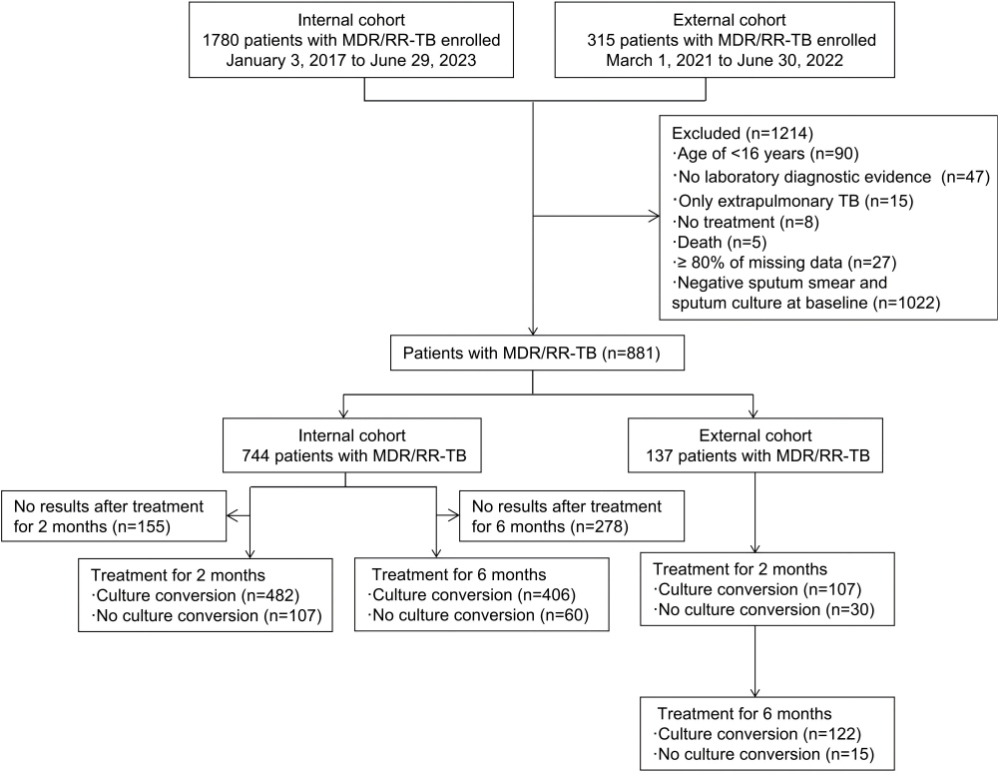
Flowchart showing patient enrollment and eligibility. MDR/RR-TB: multidrug-resistant or rifampicin-resistant tuberculosis; TB: tuberculosis.

A total of 2095 patients diagnosed with MDR/RR-TB were identified from 2 local hospitals in China. After applying strict inclusion and exclusion criteria, 42.1% (881/2095) of the patients with MDR/RR-TB were deemed eligible for the analysis. The internal cohort of 744 patients included 592 (79.6%) patients at 2 months of treatment and 466 (62.6%) patients at 6 months of treatment. Of these patients, 81.9% (485/592) achieved culture conversion after 2 months of treatment, and 87.1% (406/466) achieved culture conversion after 6 months of treatment. The external validation cohort included 137 patients, of whom 107 (78.1%) achieved culture conversion after 2 months of treatment and 122 (89.1%) achieved culture conversion after 6 months of treatment. The patient inclusion process is shown in Figure 1. As shown in Table 1, a follow-up analysis of treatment outcomes in the internal cohort indicated that, for patients with MDR/RR-TB, the OR for treatment success following culture conversion after 2 months of treatment was 8.55 (95% CI 3.31-22.08), with a sensitivity of 86.5% (95% CI 81%-90.6%) and specificity of 57.1% (95% CI 34.4%-77.4%), and the OR for treatment success following culture conversion after 6 months of treatment was 20.33 (95% CI 6.90-59.86), with a sensitivity of 92.2% (95% CI 87%-95.5%) and a specificity of 63.2% (95% CI 38.6%-82.8%).

**Table 1:** Association between early-treatment sputum culture status and treatment outcomes in patients with multidrug-resistant or rifampicin-resistant tuberculosis.

Month of treatment			Treatment outcomes				OR^a^ (95% CI)		*P* value			Sensitivity^b^ (95% CI)			Specificity^c^ (95% CI)			PPV^d^ (95% CI)			NPV^e^ (95% CI)	
			Number of failures		Number of successes																	
**2 months**										<.001			86.5 (81.0-90.6)			57.1 (34.4-77.4)			95.4 (91.1-97.7)			29.3 (16.6-45.7)
	No culture conversion	12		29		1 (reference)																
	Culture conversion	9		186		8.55 (3.31-22.08)																
**6 months**										<.001			92.2 (87.0-95.5)			63.2 (38.6-82.8)			96.0 (91.5-98.2)			46.2 (27.1-66.3)
	No culture conversion	12		14		1 (reference)																
	Culture conversion	7		166		20.33 (6.90-59.86)																

^a^OR: odds ratio.

^b^The ability of early-treatment sputum culture status to predict treatment success.

^c^The ability of early-treatment sputum culture status to predict treatment failure.

^d^PPV: positive predictive value.

^e^NPV: negative predictive value.

### Clinical Characteristics

The characteristics of patients with culture conversion and no culture conversion in the internal cohort and external validation cohort are shown in Tables S2, S3, S4, and S5 in Multimedia Appendix 1, respectively. There were statistically significant differences (*P*<.05) between patients for treatment of 2 months with culture conversion and no culture conversion in terms of age, occupation, marital status, drug resistance types, number of resistant drugs, medication compliance, pneumoconiosis, hepatitis, diabetes, cavity, mediastinal lymphadenopathy, C-reactive protein levels, lymphocyte percentage, absolute monocyte count, and sputum smear grading, whereas significant differences (*P*<.05) in age, marital status, contact individuals, TB treatment history, duration of treatment history, drug resistance types, number of resistant drugs, medication compliance, treatment regimens with LZD or BDQ, cavity, mediastinal lymphadenopathy, C-reactive protein levels, lymphocyte percentage, absolute lymphocyte count, and sputum smear grading were found between patients who achieved culture conversion and those who did not after 6 months of treatment. In the external validation cohort, patients exhibited more statistically significant differences in various characteristics between those with and without culture conversion at 2 and 6 months of treatment. After 2 months of treatment, there were no statistically significant differences in the characteristics of occupation, hepatitis, and absolute monocyte count compared to the internal cohort. However, after 6 months of treatment, there were no statistically significant differences in marital status, treatment regimens with BDQ, mediastinal lymphadenopathy, and absolute lymphocyte count compared to the internal cohort.

### Predictor Selection

In the internal cohort with 2 months of MDR/RR-TB treatment, univariate logistic regression analysis revealed that age, marital status, drug resistance types, number of resistant drugs, medication compliance, pneumoconiosis, hepatitis, diabetes, cavity, mediastinal lymphadenopathy, lymphocyte percentage, and sputum smear grading (≥3) were associated with culture conversion (Table 2). The tolerance of 12 influence factors of >0.1 and VIF of <10 suggested that they did not have multivariate collinearity (Table S6 in Multimedia Appendix 1). The Spearman correlation coefficient matrix analysis revealed a strong correlation between age and marital status, as well as between drug resistance types and the number of resistant drugs, whereas the correlation coefficients among other variables were of <0.4 (Figure S1A-C in Multimedia Appendix 1). For the internal cohort treated for 2 months, this study selected age, drug resistance pattern, medication adherence, pneumoconiosis, hepatitis, diabetes, cavity, mediastinal lymphadenopathy, lymphocyte percentage, and sputum smear grade as predictors for model development.

**Table 2:** Univariate logistic regression analysis of culture conversion in patients with multidrug-resistant or rifampicin-resistant tuberculosis after 2 months of treatment in the internal cohort.

Variable name			β (SE)		Wald *c*^2^		OR^a^ (95% CI)		*P* value
Age (y)			−0.033 (0.007)		21.721		0.967 (0.954-0.981)		*<.001* ^b^
Gender (man)			−0.167 (0.239)		0.486		0.847 (0.530-1.352)		.49
**Occupation**									
	Worker	−0.152 (0.390)		0.153		0.859 (0.400-1.845)		.70	
	Service staff	0.211 (0.358)		0.348		1.235 (0.613-2.490)		.56	
	Student	0.859 (0.558)		2.371		2.361 (0.791-7.048)		.12	
	Unemployed individual	0.442 (0.323)		1.875		1.556 (0.826-2.928)		.17	
	Others	−0.381 (0.291)		1.721		0.683 (0.386-1.207)		.19	
Ethnicity (non-Han)			0.005 (0.385)		0.000		1.006 (0.473-2.137)		.99
**Marital status**									
	Married	−1.155 (0.284)		16.594		0.315 (0.181-0.549)		*<.001* ^b^	
	Divorced or widowed	−0.759 (0.464)		2.677		0.468 (0.189-1.162)		.10	
Household registration (urban)			−0.159 (0.214)		0.555		0.853 (0.561-1.296)		.46
Current residence (urban)			−0.032 (0.214)		0.023		0.968 (0.636-1.474)		.88
BMI (kg/m^2^)			0.044 (0.029)		2.267		1.045 (0.987-1.106)		.13
Smoking habit (yes)			0.134 (0.225)		0.354		1.144 (0.735-1.778)		.55
Drinking habit (yes)			−0.123 (0.242)		0.259		0.884 (0.551-1.420)		.61
TB close contact (yes)			0.013 (0.338)		0.001		1.013 (0.522-1.965)		.97
TB treatment history (yes)			−0.160 (0.234)		0.467		0.852 (0.539-1.348)		.49
Duration of treatment history (y)			−0.015 (0.014)		1.227		0.985 (0.959-1.012)		.27
Drug resistance types (XDR^c^)			−0.904 (0.220)		16.859		0.405 (0.263-0.623)		*<.001* ^b^
Number of resistant drugs			−0.092 (0.033)		7.714		0.912 (0.854-0.973)		*.005* ^b^
Medication compliance (yes)			0.626 (0.263)		5.650		1.870 (1.116-3.132)		*.02* ^b^
Treatment regimens with LZD^d^			−0.326 (0.294)		1.228		0.722 (0.405-1.285)		.27
Treatment regimens with BDQ^e^			−0.192 (0.219)		0.770		0.825 (0.537-1.267)		.38
Treatment regimens with DLM^f^			−0.296 (0.444)		0.446		0.744 (0.312-1.774)		.50
Extrapulmonary TB (yes)			−0.008 (0.339)		0.001		0.992 (0.511-1.926)		.98
Pneumoconiosis (yes)			−1.400 (0.567)		6.099		0.247 (0.081-0.749)		*.01* ^b^
Malignant tumor (yes)			1.071 (1.043)		1.055		2.919 (0.378-22.561)		.30
Hepatitis (yes)			−1.320 (0.435)		9.201		0.267 (0.114-0.627)		*.002* ^b^
Diabetes (yes)			−0.459 (0.232)		3.901		0.632 (0.401-0.996)		*.048* ^b^
Rheumatism (yes)			−0.576 (0.537)		1.151		0.562 (0.196-1.611)		.28
Cavity (yes)			−0.727 (0.227)		10.290		0.483 (0.310-0.754)		*.001* ^b^
Calcification (yes)			−0.327 (0.265)		1.516		0.721 (0.429-1.213)		.22
Mediastinal lymphadenopathy (yes)			−1.032 (0.245)		17.765		0.356 (0.220-0.576)		*<.001* ^b^
Pleural effusion (yes)			0.290 (0.270)		1.154		1.337 (0.787-2.270)		.28
**Degree of pleural effusion**									
	Small amount	0.224 (0.283)		0.624		1.251 (0.718-2.178)		.43	
	Moderate amount	1.258 (1.040)		1.464		3.518 (0.458-26.989)		.23	
	Large amount	−0.352 (1.161)		0.092		0.704 (0.072-6.845)		.76	
**Pulmonary lesion location**									
	Upper left	1.827 (1.005)		3.302		6.214 (0.866-44.578)		.07	
	Lower left	1.350 (1.016)		1.766		3.857 (0.527-28.241)		.18	
	Upper right	1.099 (0.926)		1.408		3.000 (0.489-18.415)		.24	
	Lower middle right	1.504 (1.012)		2.210		4.500 (0.619-32.695)		.14	
	Multiple lobes or both lungs	0.562 (0.699)		0.646		1.755 (0.445-6.913)		.42	
CRP^g^ (mg/L)			−0.003 (0.002)		1.745		0.997 (0.992-1.002)		.19
Hb^h^ (g/L)			0.003 (0.005)		0.275		1.003 (0.993-1.013)		.60
Platelet count (×10^9^/L)			−0.001 (0.001)		2.547		0.999 (0.997-1.000)		.11
Lymphocyte percentage			0.037 (0.012)		10.374		1.038 (1.015-1.062)		*.001* ^b^
ALC^i^ (×10^9^/L)			0.125 (0.178)		0.492		1.133 (0.800-1.605)		.48
Monocyte percentage			0.027 (0.040)		0.457		1.027 (0.950-1.110)		.50
AMC^j^ (×10^9^/L)			−0.109 (0.179)		0.369		0.897 (0.632-1.274)		.54
Eosinophil percentage			0.074 (0.061)		1.499		1.077 (0.956-1.213)		.22
AEC^k^ (×10^9^/L)			0.196 (0.836)		0.055		1.217 (0.237-6.261)		.81
Sputum smear grading of ≥1			0.090 (0.271)		0.109		1.094 (0.643-1.860)		.74
Sputum smear grading of ≥2			−0.443 (0.299)		2.198		0.642 (0.358-1.153)		.14
Sputum smear grading of ≥3			−1.325 (0.385)		11.860		0.266 (0.125-0.565)		*<.001* ^b^
Sputum smear grading of ≥4			−0.897 (0.630)		2.025		0.408 (0.119-1.402)		.16

^a^OR: odds ratio.

^b^Variables associated with culture conversion (*P* value <.05).

^c^XDR: extensively drug resistant.

^d^LZD: linezolid.

^e^BDQ: bedaquiline.

^f^DLM: delamanid.

^g^CRP: C-reactive protein.

^h^Hb: hemoglobin.

^i^ALC: absolute lymphocyte count.

^j^AMC: absolute monocyte count.

^k^AEC: absolute eosinophil count.

In the internal cohort with 6 months of MDR/RR-TB treatment, univariate logistic regression analysis showed that age, TB treatment history, treatment regimens with LZD, treatment regimens with BDQ, cavity, sputum smear grading of ≥2, and sputum smear grading of ≥3 were associated with culture conversion (Table 3). The tolerance of 6 influence factors of >0.1 and VIF of <10 suggest that they did not have multivariate collinearity (Table S7 in Multimedia Appendix 1). The correlation coefficients between variables were of <0.4 (Figure S1D-F in Multimedia Appendix 1). For the internal cohort treated for 6 months, this study selected age, TB treatment history, treatment regimens with LZD, treatment regimens with BDQ, cavity, and sputum smear grading as predictors for model development.

**Table 3:** Univariate logistic regression analysis of culture conversion in patients with multidrug-resistant or rifampicin-resistant tuberculosis after 6 months of treatment in the internal cohort.

Variable name			β (SE)		Wald *c*^2^		OR^a^ (95% CI)		*P* value
Age (y)			−0.076 (0.021)		13.548		0.926 (0.890-0.965)		*<.001* ^b^
Gender (man)			−0.354 (0.519)		0.464		0.702 (0.254-1.941)		.50
**Occupation**									
	Worker	−0.366 (0.706)		0.269		0.693 (0.174-2.765)		.60	
	Service staff	−0.264 (0.742)		0.127		0.768 (0.179-3.287)		.72	
	Student	−0.639 (1.277)		0.250		0.528 (0.043-6.456)		.62	
	Unemployed individual	−0.134 (0.563)		0.056		0.875 (0.290-2.636)		.81	
	Others	0.290 (0.585)		0.245		1.336 (0.425-4.204)		.62	
Ethnicity (non-Han)			−0.960 (0.579)		2.751		0.383 (0.123-1.190)		.10
**Marital status**									
	Married	−0.004 (0.682)		0.000		0.996 (0.262-3.791)		>.99	
	Divorced or widowed	0.547 (0.938)		0.340		1.728 (0.275-10.854)		.56	
Household registration (urban)			0.642 (0.808)		0.631		1.901 (0.390-9.270)		.43
Current residence (urban)			−0.690 (0.748)		0.851		0.501 (0.116-2.173)		.36
BMI (kg/m^2^)			0.021 (0.061)		0.118		1.021 (0.906-1.151)		.73
Smoking habit (yes)			0.278 (0.499)		0.309		1.320 (0.496-3.511)		.58
Drinking habit (yes)			0.422 (0.518)		0.662		1.524 (0.552-4.210)		.42
TB close contact (yes)			−0.028 (0.603)		0.002		0.972 (0.298-3.169)		.96
TB treatment history (yes)			−1.466 (0.572)		6.566		0.231 (0.075-0.708)		*.01* ^b^
Duration of treatment history (y)			0.008 (0.023)		0.116		1.008 (0.964-1.054)		.73
Drug resistance types (XDR^c^)			−0.464 (0.525)		0.784		0.629 (0.225-1.758)		.38
Number of resistant drugs			−0.038 (0.269)		0.269		0.963 (0.835-1.111)		.60
Medication compliance (yes)			0.533 (0.430)		1.534		1.704 (0.733-3.959)		.22
Treatment regimens with LZD^d^			1.280 (0.439)		8.493		3.595 (1.520-8.501)		*.004* ^b^
Treatment regimens with BDQ^e^			1.351 (0.464)		8.484		3.862 (1.556-9.587)		*.004* ^b^
Treatment regimens with DLM^f^			−0.040 (1.167)		0.001		0.961 (0.098-9.462)		.97
Extrapulmonary TB (yes)			0.484 (0.636)		0.579		1.623 (0.466-5.649)		.45
Pneumoconiosis (yes)			−0.377 (0.870)		0.187		0.686 (0.125-3.773)		.67
Malignant tumor (yes)			0.905 (1.338)		0.457		2.471 (0.179-34.017)		.50
Hepatitis (yes)			0.220 (0.690)		0.101		1.246 (0.322-4.816)		.75
Diabetes (yes)			0.004 (0.420)		0.000		1.004 (0.441-2.289)		.99
Rheumatism (yes)			−0.069 (1.020)		0.005		0.934 (0.127-6.891)		.95
Cavity (yes)			−0.948 (0.450)		4.441		0.388 (0.161-0.936)		*.04* ^b^
Calcification (yes)			0.609 (0.498)		1.492		1.839 (0.692-4.884)		.22
Mediastinal lymphadenopathy (yes)			−0.070 (0.418)		0.028		0.933 (0.411-2.114)		.87
Pleural effusion (yes)			−0.132 (0.436)		0.091		0.877 (0.373-2.060)		.76
CRP^g^ (mg/L)			−0.001 (0.006)		0.046		0.999 (0.986-1.011)		.83
Hb^h^ (g/L)			−0.001 (0.011)		0.006		0.999 (0.977-1.021)		.94
Platelet count (×10^9^/L)			0.004 (0.002)		3.476		1.004 (1.000-1.008)		.06
Lymphocyte percentage			0.011 (0.043)		0.066		1.011 (0.930-1.099)		.80
ALC^i^ (×10^9^/L)			0.105 (0.592)		0.032		1.111 (0.348-3.542)		.86
Monocyte percentage			−0.137 (0.082)		2.799		0.872 (0.742-1.024)		.09
AMC^j^ (×10^9^/L)			0.985 (0.964)		1.045		2.678 (0.405-17.701)		.31
Eosinophil percentage			0.570 (0.320)		3.182		1.769 (0.945-3.309)		.07
AEC^k^ (×10^9^/L)			−7.699 (3.933)		3.832		0.000 (0.000-1.009)		.05
Sputum smear grading of ≥1			−0.499 (0.473)		1.115		0.607 (0.240-1.533)		.29
Sputum smear grading of ≥2			−1.282 (0.523)		6.020		0.277 (0.100-0.773)		*.01* ^b^
Sputum smear grading of ≥3			−1.297 (0.611)		4.510		0.273 (0.083-0.905)		*.03* ^b^
Sputum smear grading of ≥4			−1.656 (1.095)		2.287		0.191 (0.022-1.632)		.13

^a^OR: odds ratio.

^b^Variables associated with culture conversion (*P* value <.05).

^c^XDR: extensively drug resistant.

^d^LZD: linezolid.

^e^BDQ: bedaquiline.

^f^DLM: delamanid.

^g^CRP: C-reactive protein.

^h^Hb: hemoglobin.

^i^ALC: absolute lymphocyte count.

^j^AMC: absolute monocyte count.

^k^AEC: absolute eosinophil count.

### Performance Evaluation of Models

**Figure 2:**
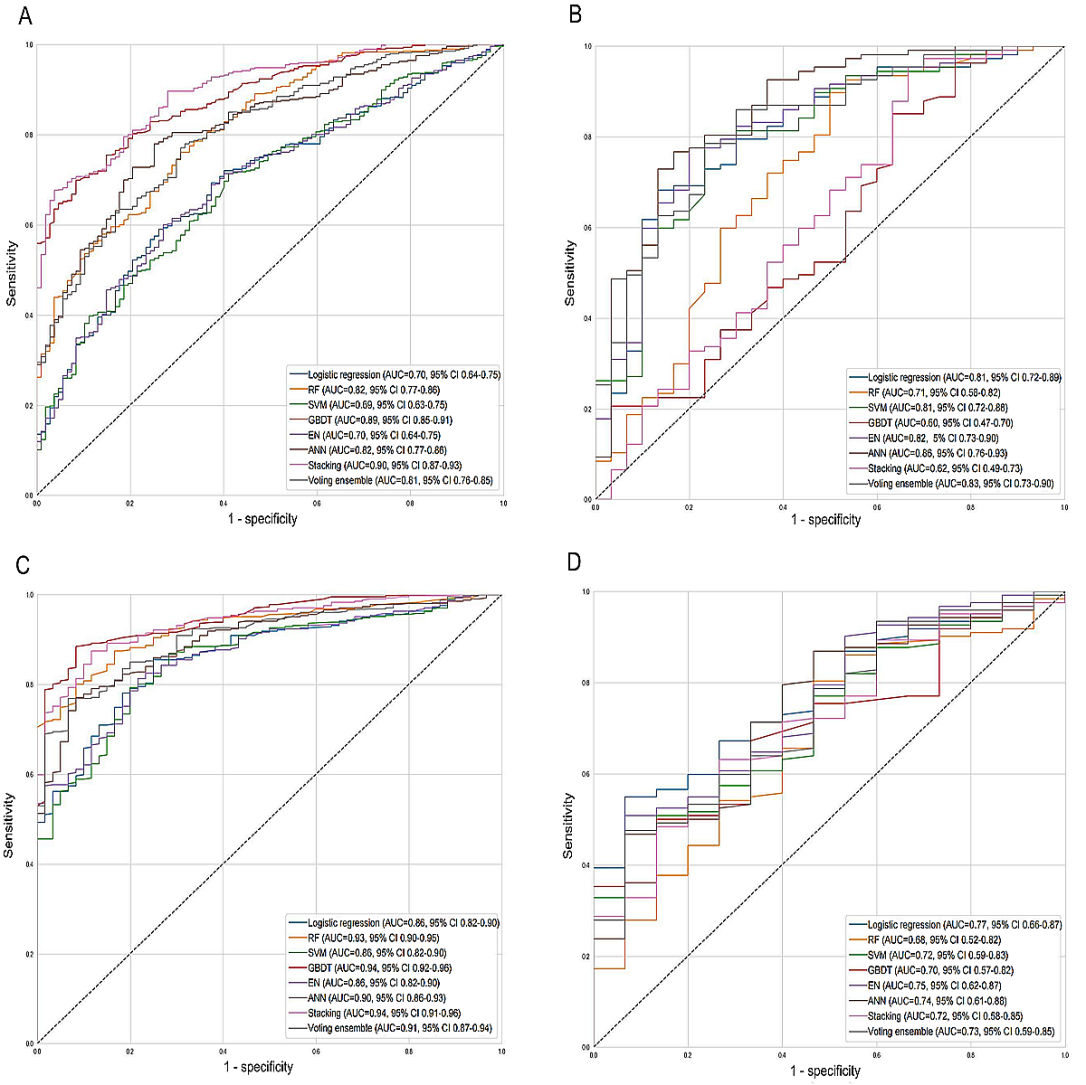
Receiver operating characteristic (ROC) curves for predicting early treatment outcomes: (A) ROC curve for predicting treatment outcomes at 2 months in the internal training cohort, (B) ROC curve for predicting treatment outcomes at 2 months in the external validation cohort, (C) ROC curve for predicting treatment outcomes at 6 months in the internal training cohort, and (D) ROC curve for predicting treatment outcomes at 6 months in the external validation cohort. ANN: artificial neural network; AUC: area under the curve; EN: elastic net; GBDT: gradient boosting decision tree; RF: random forest; SVM: support vector machine.

The metrics of performance, including the AUC, accuracy, sensitivity, specificity, and *F*_1_-score, are detailed in Table 4. For patients with MDR/RR-TB with treatment of 2 months, the AUCs for the RF, GBDT, ANN, stacking, and voting ensemble models in the training set were all of >0.80. The sensitivity and specificity of the GBDT, ANN, stacking, and voting ensemble models were close to or exceeded 0.70, with *F*_1_-scores of 0.86, 0.82, 0.91, and 0.83, respectively. In the external validation set, the AUCs for the GBDT, ANN, stacking, and voting ensemble models were 0.60, 0.86, 0.62, and 0.83, respectively. The accuracy of the ANN and voting ensemble models was 0.68 and 0.74, respectively. Although the ANN model exhibited a slightly higher AUC value than the voting ensemble model, the latter demonstrated superior accuracy. While the voting ensemble model could be considered a predictive tool, the ANN model may be preferred overall for predicting culture conversion and no culture conversion in patients with MDR/RR-TB after 2 months of treatment given its stability and clinical generalizability (Figure 2A and 2B).

**Table 4:** Performance of models predicting early treatment outcomes for patients with multidrug-resistant or rifampicin-resistant tuberculosis.

Model				AUC^a^ (95% CI)		Accuracy (95% CI)	Sensitivity (95% CI)	Specificity (95% CI)		*F*_1_-score (95% CI)
**2 months of treatment**										
	**Training set**									
		Logistic regression	0.70 (0.64-0.75)		0.65 (0.61-0.69)		0.65 (0.61-0.69)	0.64 (0.54-0.73)	0.75 (0.72-0.79)	
		RF^b^	0.82 (0.77-0.86)		0.79 (0.76-0.82)		0.83 (0.80-0.86)	0.59 (0.49-0.69)	0.86 (0.84-0.89)	
		SVM^c^	0.69 (0.63-0.75)		0.67 (0.63-0.71)		0.68 (0.64-0.72)	0.60 (0.50-0.70)	0.77 (0.74-0.80)	
		GBDT^d^	0.89 (0.85-0.91)		0.79 (0.75-0.82)		0.78 (0.75-0.82)	0.80 (0.73-0.87)	0.86 (0.83-0.88)	
		EN^e^	0.70 (0.64-0.75)		0.71 (0.67-0.75)		0.76 (0.72-0.80)	0.49 (0.40-0.58)	0.81 (0.78-0.84)	
		ANN^f^	0.82 (0.77-0.86)		0.74 (0.70-0.78)		0.74 (0.70-0.78)	0.75 (0.66-0.84)	0.82 (0.79-0.85)	
		Stacking	0.90 (0.87-0.93)		0.85 (0.82-0.88)		0.88 (0.85-0.91)	0.72 (0.63-0.80)	0.91 (0.89-0.93)	
		Voting ensemble	0.81 (0.76-0.85)		0.75 (0.71-0.79)		0.76 (0.72-0.80)	0.69 (0.60-0.78)	0.83 (0.80-0.86)	
	**Validation set**									
		Logistic regression	0.81 (0.72-0.89)		0.66 (0.57-0.73)		0.59 (0.49-0.68)	0.90 (0.79-1.00)	0.71 (0.64-0.80)	
		RF	0.71 (0.58-0.82)		0.72 (0.65-0.80)		0.78 (0.70-0.85)	0.53 (0.35-0.70)	0.81 (0.75-0.87)	
		SVM	0.81 (0.72-0.88)		0.69 (0.61-0.77)		0.67 (0.58-0.76)	0.77 (0.61-0.92)	0.77 (0.71-0.84)	
		GBDT	0.60 (0.47-0.70)		0.53 (0.45-0.61)		0.55 (0.46-0.65)	0.47 (0.27-0.64)	0.65 (0.56-0.72)	
		EN	0.82 (0.73-0.90)		0.78 (0.72-0.85)		0.78 (0.69-0.86)	0.80 (0.66-0.93)	0.85 (0.79-0.90)	
		ANN	0.86 (0.76-0.93)		0.68 (0.60-0.76)		0.63 (0.53-0.72)	0.87 (0.71-0.97)	0.75 (0.67-0.80)	
		Stacking	0.62 (0.49-0.73)		0.64 (0.55-0.71)		0.68 (0.59-0.76)	0.47 (0.27-0.64)	0.74 (0.67-0.80)	
		Voting ensemble	0.83 (0.73-0.90)		0.74 (0.66-0.80)		0.73 (0.64-0.82)	0.77 (0.60-0.90)	0.81 (0.75-0.87)	
**6 months of treatment**										
	**Training set**									
		Logistic regression	0.86 (0.82-0.90)		0.79 (0.75-0.82)		0.79 (0.75-0.82)	0.80 (0.70-0.89)	0.87 (0.84-0.89)	
		RF	0.93 (0.90-0.95)		0.86 (0.83-0.89)		0.86 (0.83-0.89)	0.83 (0.73-0.92)	0.92 (0.89-0.93)	
		SVM	0.86 (0.82-0.90)		0.79 (0.75-0.82)		0.79 (0.75-0.83)	0.78 (0.68-0.88)	0.87 (0.84-0.89)	
		GBDT	0.94 (0.92-0.96)		0.86 (0.83-0.89)		0.85 (0.82-0.89)	0.92 (0.84-0.98)	0.92 (0.89-0.93)	
		EN	0.86 (0.82-0.90)		0.82 (0.78-0.84)		0.82 (0.78-0.85)	0.77 (0.66-0.87)	0.89 (0.86-0.91)	
		ANN	0.90 (0.86-0.93)		0.80 (0.77-0.83)		0.79 (0.75-0.83)	0.87 (0.77-0.94)	0.88 (0.85-0.90)	
		Stacking	0.94 (0.91-0.96)		0.85 (0.82-0.88)		0.85 (0.82-0.88)	0.88 (0.78-0.96)	0.91 (0.89-0.93)	
		Voting ensemble	0.91 (0.87-0.94)		0.82 (0.78-0.85)		0.82 (0.78-0.85)	0.82 (0.72-0.91)	0.89 (0.86-0.91)	
	**Validation set**									
		Logistic regression	0.77 (0.66-0.87)		0.57 (0.47-0.66)		0.52 (0.43-0.62)	0.93 (0.79-1.00)	0.68 (0.60-0.76)	
		RF	0.68 (0.52-0.82)		0.64 (0.56-0.72)		0.66 (0.57-0.74)	0.53 (0.27-0.79)	0.77 (0.70-0.83)	
		SVM	0.72 (0.59-0.83)		0.55 (0.46-0.63)		0.52 (0.43-0.61)	0.73 (0.50-0.94)	0.67 (0.59-0.74)	
		GBDT	0.70 (0.57-0.82)		0.66 (0.58-0.74)		0.66 (0.57-0.74)	0.67 (0.40-0.92)	0.78 (0.71-0.83)	
		EN	0.75 (0.62-0.87)		0.61 (0.52-0.69)		0.59 (0.50-0.67)	0.73 (0.50-0.94)	0.73 (0.65-0.79)	
		ANN	0.74 (0.61-0.88)		0.72 (0.64-0.80)		0.73 (0.65-0.81)	0.60 (0.33-0.86)	0.82 (0.76-0.87)	
		Stacking	0.72 (0.58-0.85)		0.70 (0.62-0.78)		0.72 (0.64-0.80)	0.53 (0.27-0.80)	0.81 (0.75-0.87)	
		Voting ensemble	0.73 (0.59-0.85)		0.61 (0.51-0.68)		0.59 (0.50-0.67)	0.73 (0.50-0.94)	0.73 (0.65-0.79)	

^a^AUC: area under the curve.

^b^RF: random forest.

^c^SVM: support vector machine.

^d^GBDT: gradient boosting decision tree.

^e^EN: elastic net.

^f^ANN: artificial neural network.

For patients with MDR/RR-TB with treatment of 6 months, the AUCs for the logistic regression, RF, SVM, GBDT, EN, ANN, stacking, and voting ensemble models in the training set were all of >0.80 (Figure 2C). In addition, their sensitivity, specificity, and *F*_1_-scores were close to or exceeded 0.80, indicating that the 8 predictive models used in the training set demonstrated good predictive ability for culture conversion after 6 months of treatment. In the external validation set, the AUCs for the logistic regression, SVM, GBDT, EN, ANN, stacking, and voting ensemble models were all of >0.70 (Figure 2D). Furthermore, the ANN model had an accuracy, sensitivity, specificity, and *F*_1_-score of 0.72, 0.73, 0.60, and 0.82, respectively. These results suggest that the ANN model had good predictive capability for culture conversion and no culture conversion in patients with MDR/RR-TB after 6 months of treatment. The relative importance ranking of predictive factors in the models is shown in Table S8 in Multimedia Appendix 1.

## Discussion

### Principal Findings

The treatment duration for MDR/RR-TB cases is typically 20 to 24 months. Although the WHO has endorsed a standardized short-course regimen, anti-TB therapy lasts at least 6 months [3]. Prolonged treatment increases the burden on patients and health care providers, elevating the risks of poor adherence, treatment failure, and disease transmission [18]. Early prediction of treatment outcomes is crucial for timely modification of therapy regimens and improving cure rates. Sputum culture conversion is the gold standard for assessing treatment efficacy. In this study, we first developed ML models to predict early sputum culture conversion at 2 and 6 months in patients with MDR/RR-TB. We evaluated the performance of different models and identified the ANN model as the best, which can help inform clinical strategies.

Traditional early efficacy evaluation of MDR/RR-TB treatment relies on sputum smear and culture. However, sputum smear has low sensitivity and cannot distinguish between viable and nonviable bacteria [19], whereas sputum culture is susceptible to contamination and requires a prolonged turnaround time for negative results [20]. The development of predictive models for early efficacy aimed to provide timely insights into treatment outcomes for patients with MDR/RR-TB, ultimately contributing to the prevention of disease transmission. Previous research indicates that culture conversion achieved at 2 months of treatment for multidrug resistant TB serves as a reliable predictor of treatment success [21-23]. In addition, recent evidence shows that the predictive performance of culture conversion varies across follow‑up time points [13]. Specifically, culture results at months 3, 6, and 24 have been found to correlate strongly with cure rates [13,22,24]. This time‑dependent predictive value may be attributable to differences in the drug composition and combinations used in treatment regimens [25]. This study found a strong association between sputum culture status at 2 and 6 months and successful treatment outcomes in patients with MDR/RR-TB. Furthermore, the culture conversion rates at these time points in the cohort analyzed were close to or exceeded 80%, likely due to the inclusion of patients receiving regimens that incorporate new anti-TB drugs [26,27].

Several factors, including age, drug resistance types, number of resistant drugs, medication compliance, hepatitis, diabetes, cavity, and sputum smear grading, can influence treatment outcomes in patients with MDR/RR-TB [28-36]. This study revealed that these factors also influenced culture conversion after 2 months of treatment. Notably, marital status, pneumoconiosis, mediastinal lymphadenopathy, and lymphocyte percentage also serve as key determinants of culture conversion at the 2-month mark. While marital status and pneumoconiosis are nonmodifiable factors, they can serve as early prognostic markers. Among patients without culture conversion, a higher proportion of married individuals suggested a risk of household transmission, highlighting the urgent need for preventive education among spouses. Pneumoconiosis indicates compromised baseline lung function, which may lead to treatment delays or suboptimal efficacy. Clinicians who identify these features early can categorize patients as high risk, enabling proactive monitoring and intervention. In contrast, lymphocyte percentage can be readily obtained through routine laboratory tests at treatment initiation and is positively correlated with interferon gamma response [37]. Patients with higher baseline lymphocyte percentages tend to achieve earlier culture conversion, reflecting better immune status. For patients with low lymphocyte percentages, interventions such as nutritional support and immune regulation may help improve early treatment response. Furthermore, the presence of mediastinal lymphadenopathy on initial imaging often indicates more severe or disseminated disease and, thus, can inform clinical decisions regarding treatment intensity and duration.

After 6 months of treatment, factors such as age, cavity, sputum smear grading, TB treatment history, and regimens including LZD or BDQ continued to influence culture conversion, aligning with previous studies on MDR/RR-TB outcomes [31,38,39]. Age as a nonmodifiable factor can serve as a risk marker for poorer outcomes, with older patients potentially requiring closer monitoring and tailored supportive care. The presence of lung cavities and higher sputum smear grading at baseline or during treatment indicate more extensive disease and a higher bacillary burden, which may delay or impair culture conversion. It is essential for clinicians to identify these signs early and strengthen monitoring efforts accordingly. Furthermore, a history of extensive previous treatment suggests the likelihood of resistant strains and a higher risk of delayed conversion, necessitating personalized treatment adjustments and adherence support. Importantly, the inclusion of LZD or BDQ in the regimen appears to positively influence culture conversion, emphasizing the need for regimen optimization based on evolving evidence and individual patient factors. Therefore, in the early stages of treatment, inherent and immutable characteristics should be used to stratify risk and initiate proactive management. These factors can be further integrated with other relevant variables to facilitate the development of more personalized treatment strategies, ultimately aiming to improve outcomes for patients with MDR/RR-TB.

**Figure 3:**
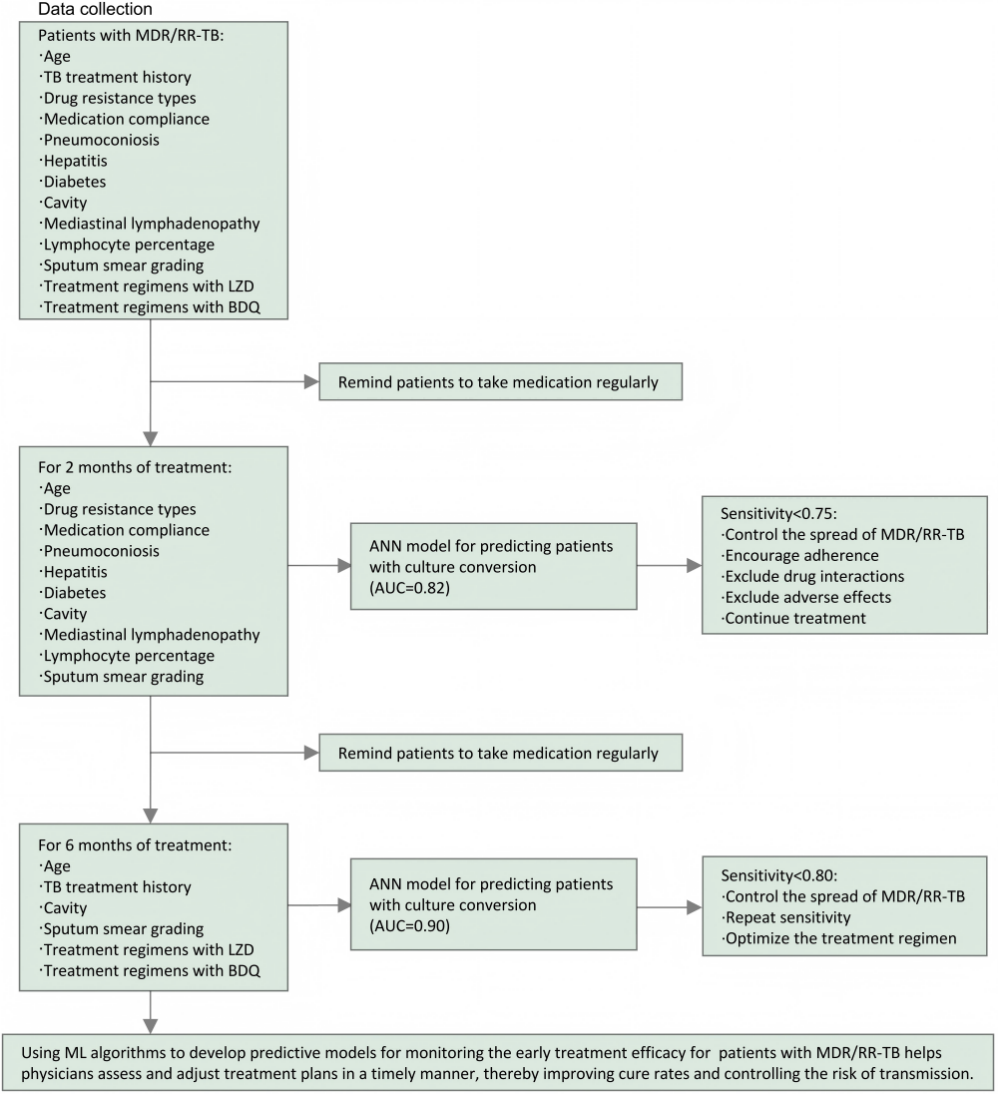
Clinical workflows for the use of machine learning (ML) algorithms to assist physicians in timely assessment of early treatment efficacy for patients with multidrug-resistant or rifampicin-resistant tuberculosis (MDR/RR-TB) and adjusting prevention and treatment plans. ANN: artificial neural network; AUC: area under the curve; BDQ: bedaquiline; LZD: linezolid; TB: tuberculosis.

For patients with MDR/RR-TB after 2 months of treatment, multiple models demonstrated good predictive performance for culture conversion in the training set. In the external validation set, the ANN model had strong generalization capabilities, with a prediction rate of 87% for patients without culture conversion. Thus, in clinical practice, the ANN model can be used to predict treatment efficacy in patients with MDR/RR-TB with 2 months of treatment. The 3 most important predictive factors identified by the ANN model were mediastinal lymphadenopathy, medication compliance, and sputum smear grading. For patients with MDR/RR-TB after 6 months of treatment, both the logistic regression and ML models showed good predictive performance regarding culture conversion during the training phase. However, in the external validation set, the ANN model performed better than the other predictive models. The 3 most important predictive factors identified by the ANN model included treatment regimens with BDQ or LZD, indicating that the use of BDQ and LZD is beneficial for culture conversion in patients with MDR/RR-TB after 6 months of treatment. In patients who meet the indications and can afford the costs, it is recommended to use treatment regimens that include BDQ or LZD. The findings of this study provide strategic guidance for the early prediction of treatment outcomes in patients with MDR/RR-TB (Figure 3).

Although our results may inform clinical practice, several uncertainties remain. The predictive framework is affected by overlapping internal and external sources of uncertainty. Internally, finite-sample estimation of model parameters yields conditional 95% CIs that may shift with larger cohorts. Moreover, the true functional relationship between predictors and culture conversion is unknown, leading to model (nonparametric) uncertainty despite the use of ensembles. Externally, the single–tertiary care setting, single‑country training cohort may limit generalizability because population differences can produce distributional drift and degrade performance. Temporal changes in treatment protocols introduce additional parametric uncertainty, and policy‑level factors act as unmeasured confounders. These sources are not directly observable in real time, challenging reliability and transportability. Recent artificial intelligence methods offer model‑agnostic, multidimensional approaches that can explicitly quantify such uncertainties. One study extended the classic suspicious-infected-death with nonpharmacological policies epidemic model into a multi-input multi-output structure and used autoregressive with exogenous input, autoregressive moving average with exogenous input, and output error system identification frameworks to learn uncertainties in real time, with promising results [40]. Future research could adopt similar techniques to dynamically estimate random or correlated uncertainties in MDR/RR-TB treatment response, yielding patient-specific probabilistic prediction intervals and thereby enhancing the robustness of early efficacy assessment.

### Comparison to Prior Work

The prediction of early treatment outcomes in MDR/RR-TB remains an unexplored area. The ANN model developed in this study demonstrated superior predictive performance compared to various models developed by Sauer et al [41], which primarily used clinical and demographic features to predict the final treatment outcomes of patients with TB. Our ANN model achieved higher AUC values, outperforming those in previous studies on TB treatment outcome prediction [42,43]. In addition, the ANN model we developed for predicting MDR/RR-TB treatment outcomes at 6 months also surpassed the clinical prediction model developed by Lv et al [44] for predicting 6-month treatment outcomes in multidrug-resistant TB. To facilitate further exploration in this field and improve clinical practice, we provide open-source tools and data for validation and application by other researchers and clinicians [45].

### Strengths and Limitations

Despite the comprehensive examination of ML algorithms for predicting early treatment outcomes in patients with MDR/RR-TB, this study has some limitations. First, it is important to note that 5 participants who died were excluded from the analysis. This decision may have introduced bias by potentially underestimating the challenges in predicting treatment outcomes among patients who are critically ill, thus impacting the overall generalizability of our model. Future studies should aim to include patients who are severely ill to validate and enhance the robustness of the model across diverse patient populations. Second, some CIs were relatively wide, primarily due to significant differences in the distribution of certain variables between the culture conversion and no–culture conversion groups, which are mainly limited by the sample size. To address this issue, future research should increase the sample size and optimize data collection. Third, the training data were sourced from a single center, necessitating the expansion to external datasets for validation to assess whether influencing factors differ in other clinical contexts. Finally, model testing was only conducted on 1 external retrospective cohort. To objectively evaluate the generalizability and reliability of the predictive model, it should be validated on multicenter independent datasets and prospective study datasets to determine its effectiveness in real clinical applications.

### Conclusions

To conclude, the ML models based on 2- and 6-month culture conversion can accurately predict treatment outcomes for patients with MDR/RR-TB. ML models, particularly the ANN model, outperformed the logistic regression model in both stability and generalizability and offer a rapid and effective tool for evaluating therapeutic efficacy at the early stages of MDR/RR-TB treatment, thereby helping control the risk of MDR/RR-TB transmission.

## Appendix

Multimedia Appendix 1 Additional tables and supplementary plots referred to in the paper.

## Abbreviations

ANN: artificial neural network
AUC: area under the curve
BDQ: bedaquiline
EN: elastic net
GBDT: gradient boosting decision tree
LZD: linezolid
MDR/RR-TB: multidrug-resistant or rifampicin-resistant tuberculosis
ML: machine learning
OR: odds ratio
RF: random forest
SVM: support vector machine
TB: tuberculosis
VIF: variance inflation factor
WHO: World Health Organization

## Data Availability

The datasets and code used in this study are publicly available and can be downloaded via the link provided in the final reference. Further analytical details generated during the study are available from the corresponding author upon reasonable request.
