# Supplementary material for: Using Machine Learning Methods to Predict Early Treatment Outcomes for Multidrug-Resistant or Rifampicin-Resistant Tuberculosis to Enhance Patient Cure Rates: Development and Validation of Multiple Models
Source: J Med Internet Res. 2025 Sep 22;27:e69998. doi: 10.2196/69998 (PMC12501533; doi:10.2196/69998)
Supplement: Multimedia Appendix 1 [file jmir_v27i1e69998_app1.doc]

**Table S1.** Optimal hyperparameters for the machine learning predictive models.

| Algorithm | Module | Optimal hyperparameters |
| --- | --- | --- |
| RF | RandomForestClassifier | max_depth=5,  min_samples_split=3,  n_estimators=15 |
| SVM | SVC | kernel='linear',  C=0.1 |
| GBDT | GradientBoostingClassifier | max_depth=5,  min_samples_split=3,  n_estimators=17 |
| EN | LogisticRegression | C=0.1  l1_ratio=0.5 |
| ANN | MLPClassifier | hidden_layer_sizes=(20, 20),  learning_rate= 0.001,  Activation_function=relu |

Abbreviations: RF, random forest; SVM, support vector machine; GBDT, gradient boosting decision tree; EN, elastic net; ANN, artificial neural network.

**Table S2. Sociodemographic and clinical characteristics of MDR/RR-TB patients with 2 months of treatment in internal cohort.**

| **Characteristics** | **Culture conversion (n=485)** |  | **No culture conversion (n=107)** | **χ^2^/t/z** | ***P*** |
| --- | --- | --- | --- | --- | --- |
|  | **n (%)/mean±SD/median (IQR)** |  | **n (%)/mean±SD/median (IQR)** |  |  |
| Age (years) | 37.0 (25.0, 75.0) |  | 50.0 (36.0, 56.0) | -5.054 | **<0.001** |
| Gender |  |  |  | 0.487 | 0.485 |
| Man | 337 (69.5) |  | 78 (72.9) |  |  |
| Woman | 148 (30.5) |  | 29 (27.1) |  |  |
| Occupation |  |  |  | 29.528 | **<0.001** |
| Farmer | 144 (29.8) |  | 34 (31.8) |  |  |
| Worker | 40 (8.2) |  | 11 (10.3) |  |  |
| Service staff | 68 (14.0) |  | 13 (12.1) |  |  |
| Student | 40 (8.2) |  | 4 (3.7) |  |  |
| Unemployed individual | 112 (23.1) |  | 17 (15.9) |  |  |
| Others | 81 (16.7) |  | 28 (26.2) |  |  |
| Ethnicity |  |  |  | 0.000 | 0.989 |
| Han ethnicity | 444 (91.5) |  | 98 (91.6) |  |  |
| Non-Han ethnicity | 41 (8.5) |  | 9 (8.4) |  |  |
| Marital status |  |  |  | 18.022 | **<0.001** |
| Single | 177 (36.5) |  | 17 (15.9) |  |  |
| Married | 269 (55.5) |  | 82 (76.6) |  |  |
| Divorced or widowed | 3 9(8.0) |  | 8 (7.5) |  |  |
| Household registration |  |  |  | 0.556 | 0.456 |
| Rural | 255 (52.6) |  | 52 (48.6) |  |  |
| Urban | 230 (47.4) |  | 55 (51.4) |  |  |
| Current residence |  |  |  | 0.023 | 0.880 |
| Rural | 226 (46.6) |  | 49 (45.8) |  |  |
| Urban | 259 (53.4) |  | 58 (54.2) |  |  |
| BMI（kg/m^2^） | 20.76 (18.21, 23.44) |  | 19.95 (17.58, 23.12) | -1.724 | 0.085 |
| Smoking habit |  |  |  | 0.355 | 0.551 |
| No | 307 (63.3) |  | 71 (66.4) |  |  |
| Yes | 178 (36.7) |  | 36 (33.6) |  |  |
| Quit smoking |  |  |  | 0.243 | 0.622 |
| No | 97 (20.0) |  | 18 (16.8) |  |  |
| Yes | 81 (16.7) |  | 18 (16.8) |  |  |
| Drinking habit |  |  |  | 0.259 | 0.611 |
| No | 365 (75.3) |  | 78 (72.9) |  |  |
| Yes | 120 (24.7) |  | 29 (27.1) |  |  |
| Quit drinking |  |  |  | 0.003 | 0.958 |
| No | 78 (16.1) |  | 19 (17.8) |  |  |
| Yes | 42 (8.7) |  | 10 (9.3) |  |  |
| TB close contact |  |  |  | 0.001 | 0.970 |
| No | 430 (88.7) |  | 95 (88.8) |  |  |
| Yes | 55 (11.3) |  | 12 (11.2) |  |  |
| Contact subjects |  |  |  | 2.520 | 0.211 |
| Relative | 38 (7.8) |  | 10 (9.3) |  |  |
| Friend | 15 (3.1) |  | 1 (0.9) |  |  |
| Colleague | 2 (0.4) |  | 1 (0.9) |  |  |
| TB treatment history |  |  |  | 0.467 | 0.494 |
| No | 157 (32.4) |  | 31 (29.0) |  |  |
| Yes | 328 (67.6) |  | 76 (71.0) |  |  |
| Duration of treatment history (years) | 1.0 (0.0,5.0) |  | 1.0 (0.0, 6.0) | -0.680 | 0.497 |
| Drug resistance types |  |  |  | 26.198 | **<0.001** |
| RR | 26 (5.4) |  | 11 (10.3) |  |  |
| MDR | 263 (54.2) |  | 29 (27.1) |  |  |
| XDR | 196 (40.4) |  | 67 (62.6) |  |  |
| Number of resistant drugs | 4.0 (2.0,6.0) |  | 6.0 (3.0,9.0) | -2.437 | **0.015** |
| Medication compliance |  |  |  | 5.780 | **0.016** |
| No | 68 (14.0) |  | 25 (23.4) |  |  |
| Yes | 417 (86.0) |  | 82 (76.6) |  |  |
| Treatment regimens |  |  |  |  |  |
| Without LZD | 95 (19.6) |  | 16 (15.0) | 1.236 | 0.266 |
| With LZD | 390 (80.4) |  | 91 (85.0) |  |  |
| Without BDQ | 312 (64.3) |  | 64 (59.8) | 0.772 | 0.380 |
| Witht BDQ | 173 (35.7) |  | 43 (40.2) |  |  |
| Without DLM | 461 (95.1) |  | 100 (93.5) | 0.449 | 0.503 |
| With DLM | 24 (4.9) |  | 7 (6.5) |  |  |
| Comorbidity |  |  |  |  |  |
| Extrapulmonary TB |  |  |  | 0.001 | 0.981 |
| No | 431 (88.9) |  | 95 (88.8) |  |  |
| Yes | 54 (11.1) |  | 12 (11.2) |  |  |
| Pneumoconiosis |  |  |  | 5.271 | **0.022** |
| No | 478 (98.6) |  | 101 (94.4) |  |  |
| Yes | 7 (1.4) |  | 6 (5.6) |  |  |
| Malignant tumor |  |  |  | 0.525 | 0.469 |
| No | 472 (97.3) |  | 106 (99.1) |  |  |
| Yes | 13 (2.7) |  | 1 (0.9) |  |  |
| Hepatitis |  |  |  | 8.721 | **0.003** |
| No | 472 (97.3) |  | 97 (90.7) |  |  |
| Yes | 13 (2.7) |  | 10 (9.3) |  |  |
| Diabetes |  |  |  | 3.944 | **0.047** |
| No | 371 (76.5) |  | 72 (67.3) |  |  |
| Yes | 114 (23.5) |  | 35 (32.7) |  |  |
| HIV |  |  |  | NA | 1.000 |
| No | 484 (99.8) |  | 107 (100.0) |  |  |
| Yes | 1 (0.2) |  | 0 (0.0) |  |  |
| Rheumatism |  |  |  | 0.601 | 0.438 |
| No | 472 (97.3) |  | 102 (95.3) |  |  |
| Yes | 13 (2.7) |  | 5 (4.7) |  |  |
| Cavity |  |  |  | 10.559 | **0.001** |
| No | 238 (49.1) |  | 34 (31.8) |  |  |
| Yes | 247 (50.9) |  | 73 (68.2) |  |  |
| Calcification |  |  |  | 1.525 | 0.217 |
| No | 405 (83.5) |  | 84 (78.5) |  |  |
| Yes | 80 (16.5) |  | 23 (21.5) |  |  |
| Mediastinal lymphadenopathy |  |  |  | 18.785 | **<0.001** |
| No | 416 (85.8) |  | 73 (68.2) |  |  |
| Yes | 69 (14.2) |  | 34 (31.8) |  |  |
| Pleural effusion |  |  |  | 1.160 | 0.281 |
| No | 371 (76.5) |  | 87 (81.3) |  |  |
| Yes | 114 (23.5) |  | 20 (18.7) |  |  |
| Degree of pleural effusion |  |  |  | 2.303 | 0.479 |
| No effusion | 371 (76.5) |  | 87 (81.3) |  |  |
| Small amount | 96 (19.8) |  | 18 (16.9) |  |  |
| Moderate amount | 15 (3.1) |  | 1 (0.9) |  |  |
| Large amount | 3 (0.6) |  | 1 (0.9) |  |  |
| Pulmonary lesion location |  |  |  | 6.838 | 0.213 |
| No lesions | 7 (1.4) |  | 3 (2.8) |  |  |
| Upper left | 29 (6.0) |  | 2 (1.9) |  |  |
| Lower left | 18 (3.7) |  | 2 (1.9) |  |  |
| Upper right | 21 (4.3) |  | 3 (2.8) |  |  |
| Lower middle right | 21 (4.3) |  | 2 (1.9) |  |  |
| Multiple lobes or both lung | 389 (80.2) |  | 95 (88.8) |  |  |
| CRP (mg/L) | 12.45 (2.15, 41.63) |  | 16.57 (5.33, 53.20) | -2.584 | **0.010** |
| Hb (g/L) | 126.80 ± 21.35 |  | 125.61 ± 21.24 | -0.524 | 0.601 |
| Platelet(×10^9^/L) | 264.00 (207.50, 331.00) |  | 284.00 (214.00, 364.00) | -1.282 | 0.200 |
| Lymphocyte percentage (%) | 22.90 (15.95, 30.60) |  | 20.21 ± 8.65 | -3.161 | **0.002** |
| ALC(×10^9^/L) | 1.45 (1.11, 1.87) |  | 1.50 ± 0.56 | -0.389 | 0.697 |
| Monocyte percentage (%) | 8.00 (6.50, 10.00) |  | 7.60 (6.10, 9.70) | -1.176 | 0.240 |
| AMC (×10^9^/L) | 0.55 (0.41, 0.70) |  | 0.59 (0.46, 0.74) | -1.993 | **0.046** |
| Eosinophil percentage (%) | 1.80 (1.00, 3.10) |  | 1.60 (1.00, 2.70) | -1.029 | 0.303 |
| AEC (×10^9^/L) | 0.12 (0.07, 0.20) |  | 0.13 (0.08, 0.19) | -0.646 | 0.518 |
| Sputum smear grading |  |  |  | 16.645 | **0.002** |
| No AFB | 182 (37.5) |  | 33 (30.9) |  |  |
| 1+ | 187 (38.6) |  | 31 (29.0) |  |  |
| 2+ | 85 (17.5) |  | 24 (22.4) |  |  |
| 3+ | 22 (4.5) |  | 15 (14.0) |  |  |
| 4+ | 9 (1.9) |  | 4 (3.7) |  |  |

Abbreviations: TB, tuberculosis; RR, rifampicin-resistant; MDR, multidrug-resistant; XDR, extensively drug-resistant; LZD, linezolid; BDQ, bedaquiline; DLM, delamanid; BMI, body mass index; HIV, human immunodeficiency virus; CRP, C-reactive protein; Hb, hemoglobin; ALC, absolute lymphocyte count; AMC, absolute monocyte count; AEC, absolute eosinophil count; AFB, acid-fast bacilli. SD, standard deviation; IQR, interquartile range; NA, not applicable.

**Table S3. Sociodemographic and clinical characteristics of MDR/RR-TB patients with 6 months of treatment in internal cohort.**

| **Characteristics** | **Culture conversion (*n*=406)** |  | **No culture conversion (*n*=60)** | **χ^2^/t/z** | ***P*** |
| --- | --- | --- | --- | --- | --- |
|  | **n (%)/mean±SD/median (IQR)** |  | **n (%)/mean±SD/median (IQR)** |  |  |
| Age (years) | 39.0 (27.0, 52.0) |  | 52.75 ± 13.50 | -5.762 | **<0.001** |
| Gender |  |  |  | 2.693 | 0.101 |
| Man | 283 (69.7) |  | 48 (80.0) |  |  |
| Woman | 123 (30.3) |  | 12 (20.0) |  |  |
| Occupation |  |  |  | 10.513 | 0.062 |
| Farmer | 115 (28.3) |  | 18 (30.0) |  |  |
| Worker | 36 (8.9) |  | 6 (10.0) |  |  |
| Service staff | 60 (14.8) |  | 6 (10.0) |  |  |
| Student | 33 (8.1) |  | 1 (1.7) |  |  |
| Unemployed individual | 92 (22.7) |  | 10 (16.7) |  |  |
| Others | 70 (17.2) |  | 19 (31.7) |  |  |
| Ethnicity |  |  |  | 0.591 | 0.442 |
| Han ethnicity | 371 (91.4) |  | 53 (88.3) |  |  |
| Non-Han ethnicity | 35 (8.6) |  | 7 (11.7) |  |  |
| Marital status |  |  |  | 12.442 | **0.002** |
| Single | 139 (34.2) |  | 7 (11.7) |  |  |
| Married | 239 (58.9) |  | 48 (80.0) |  |  |
| Divorced or widowed | 28 (6.9) |  | 5 (8.3) |  |  |
| Household registration |  |  |  | 0.023 | 0.880 |
| Rural | 214 (52.7) |  | 31 (51.7) |  |  |
| Urban | 192 (47.3) |  | 29 (48.3) |  |  |
| Current residence |  |  |  | 0.003 | 0.956 |
| Rural | 191 (47.0) |  | 28 (46.7) |  |  |
| Urban | 215 (53.0) |  | 32 (53.3) |  |  |
| BMI（kg/m^2^） | 21.14 ± 3.75 |  | 20.91 ± 3.81 | -0.436 | 0.663 |
| Smoking habit |  |  |  | 0.308 | 0.579 |
| No | 252 (62.1) |  | 35 (58.3) |  |  |
| Yes | 154 (37.9) |  | 25 (41.7) |  |  |
| Quit smoking |  |  |  | 0.066 | 0.798 |
| No | 82 (20.2) |  | 14 (23.3) |  |  |
| Yes | 72 (17.7) |  | 11 (18.3) |  |  |
| Drinking habit |  |  |  | 0.133 | 0.715 |
| No | 300 (73.9) |  | 43 (71.7) |  |  |
| Yes | 106 (26.1) |  | 17 (28.3) |  |  |
| Quit drinking |  |  |  | 0.727 | 0.394 |
| No | 70 (17.2) |  | 13 (21.7) |  |  |
| Yes | 36 (8.9) |  | 4 (6.7) |  |  |
| TB close contact |  |  |  | 0.265 | 0.607 |
| No | 356 (87.7) |  | 54 (90.0) |  |  |
| Yes | 50 (12.3) |  | 6 (10.0) |  |  |
| Contact subjects |  |  |  | 7.401 | **0.022** |
| Relative | 35 (8.6) |  | 4 (6.7) |  |  |
| Friend | 14 (3.4) |  | 0 (0.0) |  |  |
| Colleague | 1 (0.2) |  | 2 (3.3) |  |  |
| TB treatment history |  |  |  | 13.623 | **<0.001** |
| No | 136 (33.5) |  | 6 (10.0) |  |  |
| Yes | 270 (66.5) |  | 54 (90.0) |  |  |
| Duration of treatment history (years) | 1.0 (0.0, 4.0) |  | 5.0 (1.0, 10.8) | -4.382 | **<0.001** |
| Drug resistance types |  |  |  | 18.510 | **<0.001** |
| RR | 22 (5.4) |  | 6 (10.0) |  |  |
| MDR | 201 (49.5) |  | 12 (20.0) |  |  |
| XDR | 183 (45.1) |  | 42 (70.0) |  |  |
| Number of resistant drugs | 5.0 (3.0, 7.0) |  | 6.5 (4.0,9.0) | -3.022 | **0.003** |
| Medication compliance |  |  |  | 11.013 | **<0.001** |
| No | 59 (14.5) |  | 19 (31.7) |  |  |
| Yes | 347 (85.5) |  | 41 (68.3) |  |  |
| Treatment regimens |  |  |  |  |  |
| Without LZD | 67 (16.5) |  | 18 (30.0) | 6.386 | **0.012** |
| With LZD | 339 (83.5) |  | 42 (70.0) |  |  |
| Without BDQ | 233 (57.4) |  | 51 (85.0) | 16.743 | **<0.001** |
| Witht BDQ | 173 (42.6) |  | 9 (15.0) |  |  |
| Without DLM | 378 (93.1) |  | 59 (98.3) | 1.636 | 0.201 |
| With DLM | 28 (6.9) |  | 1 (1.7) |  |  |
| Comorbidity |  |  |  |  |  |
| Extrapulmonary TB |  |  |  | 0.183 | 0.669 |
| No | 365 (89.9) |  | 55 (91.7) |  |  |
| Yes | 41 (10.1) |  | 5 (8.3) |  |  |
| Pneumoconiosis |  |  |  | 2.352 | 0.125 |
| No | 397 (97.8) |  | 56 (93.3) |  |  |
| Yes | 9 (2.2) |  | 4 (6.7) |  |  |
| Malignant tumor |  |  |  | 0.000 | 1.000 |
| No | 396 (97.5) |  | 59 (98.3) |  |  |
| Yes | 10 (2.5) |  | 1 (1.7) |  |  |
| Hepatitis |  |  |  | 3.476 | 0.062 |
| No | 391 (96.3) |  | 54 (90.0) |  |  |
| Yes | 15 (3.7) |  | 6 (10.0) |  |  |
| Diabetes |  |  |  | 3.236 | 0.072 |
| No | 302 (74.4) |  | 38 (63.3) |  |  |
| Yes | 104 (25.6) |  | 22 (36.7) |  |  |
| HIV |  |  |  | NA | 1.000 |
| No | 405 (99.8) |  | 60 (100.0) |  |  |
| Yes | 1 (0.2) |  | 0 (0.0) |  |  |
| Rheumatism |  |  |  | 0.000 | 1.000 |
| No | 392 (96.6) |  | 58 (96.7) |  |  |
| Yes | 14 (3.4) |  | 2 (3.3) |  |  |
| Cavity |  |  |  | 17.829 | **<0.001** |
| No | 192 (47.3) |  | 11 (18.3) |  |  |
| Yes | 214 (52.7) |  | 49 (81.7) |  |  |
| Calcification |  |  |  | 0.005 | 0.943 |
| No | 330 (81.3) |  | 49 (81.7) |  |  |
| Yes | 76 (18.7) |  | 11 (18.3) |  |  |
| Mediastinal lymphadenopathy |  |  |  | 13.308 | **<0.001** |
| No | 338 (83.3) |  | 38 (63.3) |  |  |
| Yes | 68 (16.7) |  | 22 (36.7) |  |  |
| Pleural effusion |  |  |  | 0.412 | 0.521 |
| No | 313 (77.1) |  | 44 (73.3) |  |  |
| Yes | 93 (22.9) |  | 16 (26.7) |  |  |
| Degree of pleural effusion |  |  |  | 2.906 | 0.374 |
| No effusion | 313 (77.1) |  | 44 (73.3) |  |  |
| Small amount | 79 (19.5) |  | 16 (26.7) |  |  |
| Moderate amount | 12 (3.0) |  | 0 (0.0) |  |  |
| Large amount | 2 (0.5) |  | 0 (0.0) |  |  |
| Pulmonary lesion location |  |  |  | 8.070 | 0.106 |
| No lesions | 3 (0.7) |  | 2 (3.3) |  |  |
| Upper left | 22 (5.4) |  | 0 (0.0) |  |  |
| Lower left | 12 (3.0) |  | 0 (0.0) |  |  |
| Upper right | 15 (3.7) |  | 2 (3.3) |  |  |
| Lower middle right | 13 (3.2) |  | 2 (3.3) |  |  |
| Multiple lobes or both lung | 341 (84.0) |  | 54 (90.0) |  |  |
| CRP (mg/L) | 11.33 (2.11, 38.90) |  | 18.77 (8.52,55.77) | -2.868 | **0.004** |
| Hb (g/L) | 128.00 (113.00, 142.00) |  | 124.88 ± 22.22 | -0.838 | 0.402 |
| Platelet(×10^9^/L) | 259.00 (204.00, 328.25) |  | 257.00 (179.00, 309.50) | -1.318 | 0.188 |
| Lymphocyte percentage (%) | 23.45 (16.00, 30.83) |  | 18.15 (13.78, 26.25) | -2.915 | **0.004** |
| ALC(×10^9^/L) | 1.47 (1.11, 1.91) |  | 1.30 (0.91, 1.80) | -2.314 | **0.021** |
| Monocyte percentage (%) | 7.80 (6.20, 9.60) |  | 8.25 (6.50, 10.08) | -1.027 | 0.305 |
| AMC (×10^9^/L) | 0.53 (0.40, 0.68) |  | 0.57 (0.47, 0.81) | -1.911 | 0.056 |
| Eosinophil percentage (%) | 1.90 (1.10, 3.20) |  | 2.05 (1.10, 3.20) | -0.192 | 0.848 |
| AEC (×10^9^/L) | 0.12 (0.07, 0.20) |  | 0.15 (0.09, 0.24) | -1.426 | 0.154 |
| Sputum smear grading |  |  |  | 15.568 | **0.003** |
| No AFB | 159 (39.2) |  | 16 (26.7) |  |  |
| 1+ | 151 (37.2) |  | 16 (26.7) |  |  |
| 2+ | 67 (16.5) |  | 16 (26.7) |  |  |
| 3+ | 24 (5.9) |  | 10 (16.7) |  |  |
| 4+ | 5 (1.2) |  | 2 (3.3) |  |  |

Abbreviations: TB, tuberculosis; RR, rifampicin-resistant; MDR, multidrug-resistant; XDR, extensively drug-resistant; LZD, linezolid; BDQ, bedaquiline; DLM, delamanid; BMI, body mass index; HIV, human immunodeficiency virus; CRP, C-reactive protein; Hb, hemoglobin; ALC, absolute lymphocyte count; AMC, absolute monocyte count; AEC, absolute eosinophil count; AFB, acid-fast bacilli. SD, standard deviation; IQR, interquartile range; NA, not applicable.

**Table S4. Testing for collinearity among selected predictors for MDR/RR-TB patients with 2 months of treatment.**

| **Predictors** | **Tolerance** | **VIF** |
| --- | --- | --- |
| Age | 0.561 | 1.784 |
| Marital status | 0.673 | 1.486 |
| Drug resistance types | 0.495 | 2.019 |
| Number of resistant drugs | 0.520 | 1.921 |
| Medication compliance | 0.971 | 1.030 |
| Pneumoconiosis | 0.925 | 1.081 |
| Hepatitis | 0.955 | 1.047 |
| Diabetes | 0.845 | 1.184 |
| Cavity | 0.868 | 1.153 |
| Mediastinal lymphadenopathy | 0.799 | 1.251 |
| Lymphocyte percentage | 0.925 | 1.081 |
| Sputum smear grading | 0.933 | 1.072 |

Abbreviations: VIF, Variance inflation factor.

Table S5. Testing for collinearity among selected predictors for MDR/RR-TB patients with 6 months of treatment.

| **Predictors** | **Tolerance** | **VIF** |
| --- | --- | --- |
| Age | 0.952 | 1.050 |
| TB treatment history | 0.961 | 1.040 |
| Treatment regimens with LZD | 0.916 | 1.091 |
| Treatment regimens with BDQ | 0.937 | 1.067 |
| Cavity | 0.911 | 1.097 |
| Sputum smear grading | 0.978 | 1.023 |

Abbreviations: VIF, Variance inflation factor; LZD, linezolid; BDQ, bedaquiline.

**Figure S1.** Analysis of spearman correlation coefficient matrix among variables in internal cohort. (A) Analysis of spearman correlation coefficient matrix for 2 months of treatment. (B) Analysis of spearman correlation coefficient matrix for culture conversion after 2 months of treatment. (C) Analysis of spearman correlation coefficient matrix for no culture conversion after 2 months of treatment. (D) Analysis of spearman correlation coefficient matrix for 6 months of treatment. (E) Analysis of spearman correlation coefficient matrix for culture conversion after 6 months of treatment. (F) Analysis of spearman correlation coefficient matrix for no culture conversion after 6 months of treatment.

**Table S6. Sociodemographic and clinical characteristics of MDR/RR-TB patients with 2 months of treatment in external cohort.**

| **Characteristics** | **Culture conversion (**n=107) |  | **No culture conversion (**n=30) | **χ^2^**/z | ***P*** |
| --- | --- | --- | --- | --- | --- |
|  | **n (%)/mean±SD/median (IQR)** |  | **n (%)/mean±SD/median (IQR)** |  |  |
| Age (years) | 39.0 (26.0, 55.0) |  | 53.63 ± 14.24 | -3.418 | **<0.001** |
| Gender |  |  |  | 9.573 | **0.002** |
| Man | 64 (59.8) |  | 27 (90.0) |  |  |
| Woman | 43 (40.2) |  | 3 (10.0) |  |  |
| Occupation |  |  |  | 7.701 | 0.103 |
| Farmer | 0 (0.0) |  | 0 (0.0) |  |  |
| Worker | 15 (14.0) |  | 8 (26.7) |  |  |
| Service staff | 4 (3.7) |  | 3 (10.0) |  |  |
| Student | 7 (6.6) |  | 0 (0.0) |  |  |
| Unemployed individual | 23 (21.5) |  | 8 (26.7) |  |  |
| Others | 58 (54.2) |  | 11 (36.7) |  |  |
| Ethnicity |  |  |  | NA | NA |
| Han ethnicity | 107 (100.0) |  | 30 (100.0) |  |  |
| Non-Han ethnicity | 0 (0) |  | 0 (0.0) |  |  |
| Marital status |  |  |  | 6.034 | **0.028** |
| Single | 33 (30.9) |  | 3 (10.0) |  |  |
| Married | 73 (68.2) |  | 27 (90.0) |  |  |
| Divorced or widowed | 1 (0.9) |  | 0 (0.0) |  |  |
| Household registration |  |  |  | 5.067 | **0.024** |
| Rural | 43 (40.2) |  | 19 (63.3) |  |  |
| Urban | 64 (59.8) |  | 11 (36.7) |  |  |
| Current residence |  |  |  | 5.067 | **0.024** |
| Rural | 43 (40.2) |  | 19 (63.3) |  |  |
| Urban | 64 (59.8) |  | 11 (36.7) |  |  |
| BMI（kg/m^2^） | 19.49 (17.94, 20.62) |  | 18.37 (17.04, 21.47) | -0.940 | 0.347 |
| Smoking habit |  |  |  | 4.264 | **0.039** |
| No | 72 (67.3) |  | 14 (46.7) |  |  |
| Yes | 35 (32.7) |  | 16 (53.3) |  |  |
| Quit smoking |  |  |  | 0.130 | 0.718 |
| No | 15 (14.0) |  | 6 (20.0) |  |  |
| Yes | 20 (18.7) |  | 10 (33.3) |  |  |
| Drinking habit |  |  |  | 11.554 | **<0.001** |
| No | 99 (92.5) |  | 20 (66.7) |  |  |
| Yes | 8 (7.5) |  | 10 (33.3) |  |  |
| Quit drinking |  |  |  | 0.000 | 1.000 |
| No | 4 (3.7) |  | 6 (20.0) |  |  |
| Yes | 4 (3.7) |  | 4 (13.3) |  |  |
| TB close contact |  |  |  | NA | 1.000 |
| No | 106 (99.1) |  | 30 (100.0) |  |  |
| Yes | 1 (0.9) |  | 0 (0.0) |  |  |
| Contact subjects |  |  |  | NA | NA |
| Relative | 1 (0.9) |  | 0 (0.0) |  |  |
| Friend | 0 (0.0) |  | 0 (0.0) |  |  |
| Colleague | 0 (0.0) |  | 0 (0.0) |  |  |
| TB treatment history |  |  |  | 2.872 | 0.090 |
| No | 43 (40.2) |  | 7 (23.3) |  |  |
| Yes | 64 (59.8) |  | 23 (76.7) |  |  |
| Duration of treatment history (years) | 0.3 (0.0, 0.7) |  | 0.5 (0.2, 2.0) | -3.185 | **0.001** |
| Drug resistance types |  |  |  | 17.167 | **<0.001** |
| RR | 18 (16.8) |  | 0 (0.0) |  |  |
| MDR | 62 (58.0) |  | 11 (36.7) |  |  |
| XDR | 27 (25.2) |  | 19 (63.3) |  |  |
| Number of resistant drugs | 2.0 (2.0, 4.0) |  | 3.0 (2.0, 4.0) | -3.378 | **<0.001** |
| Medication compliance |  |  |  | 41.404 | **<0.001** |
| No | 1 (0.9) |  | 13 (43.3) |  |  |
| Yes | 106 (99.1) |  | 17 (56.7) |  |  |
| Treatment regimens |  |  |  |  |  |
| Without LZD | 6 (5.6) |  | 6 (20.0) | 4.406 | **0.036** |
| With LZD | 101 (94.4) |  | 24 (80.0) |  |  |
| Without BDQ | 75 (70.1) |  | 23 (76.7) | 0.497 | 0.481 |
| Witht BDQ | 32 (29.9) |  | 7 (23.3) |  |  |
| Without DLM | 101 (94.4) |  | 23 (76.7) | 6.632 | **0.010** |
| With DLM | 6 (5.6) |  | 7 (23.3) |  |  |
| Comorbidity |  |  |  |  |  |
| Extrapulmonary TB |  |  |  | 1.504 | 0.220 |
| No | 98 (91.6) |  | 30 (100.0) |  |  |
| Yes | 9 (8.4) |  | 0 (0.0) |  |  |
| Pneumoconiosis |  |  |  | 7.021 | **0.008** |
| No | 106 (99.1) |  | 26 (86.7) |  |  |
| Yes | 1 (0.9) |  | 4 (13.3) |  |  |
| Malignant tumor |  |  |  | NA | 1.000 |
| No | 105 (98.1) |  | 30 (100.0) |  |  |
| Yes | 2 (1.9) |  | 0 (0.0) |  |  |
| Hepatitis |  |  |  | 1.626 | 0.202 |
| No | 102 (95.3) |  | 26 (86.7) |  |  |
| Yes | 5 (4.7) |  | 4 (13.3) |  |  |
| Diabetes |  |  |  | 21.031 | **<0.001** |
| No | 83 (77.6) |  | 10 (33.3) |  |  |
| Yes | 24 (22.4) |  | 20 (66.7) |  |  |
| HIV |  |  |  | NA | NA |
| No | 107 (100.0) |  | 30 (100.0) |  |  |
| Yes | 0 (0.0) |  | 0 (0.0) |  |  |
| Rheumatism |  |  |  | NA | 1.000 |
| No | 106 (99.1) |  | 30 (100.0) |  |  |
| Yes | 1 (0.9) |  | 0 (0.0) |  |  |
| Cavity |  |  |  | 12.472 | **<0.001** |
| No | 44 (41.1) |  | 2 (6.7) |  |  |
| Yes | 63 (58.9) |  | 28 (93.3) |  |  |
| Calcification |  |  |  | 3.715 | 0.054 |
| No | 98 (91.6) |  | 23 (76.7) |  |  |
| Yes | 9 (8.4) |  | 7 (23.3) |  |  |
| Mediastinal lymphadenopathy |  |  |  | 25.412 | **<0.001** |
| No | 84 (78.5) |  | 9 (30.0) |  |  |
| Yes | 23 (21.5) |  | 21 (70.0) |  |  |
| Pleural effusion |  |  |  | NA | NA |
| No | 107 (100.0) |  | 30 (100.0) |  |  |
| Yes | 0 (0.0) |  | 0 (0.0) |  |  |
| Degree of pleural effusion |  |  |  | NA | NA |
| No effusion | 107 (100.0) |  | 30 (100.0) |  |  |
| Small amount | 0 (0.0) |  | 0 (0.0) |  |  |
| Moderate amount | 0 (0.0) |  | 0 (0.0) |  |  |
| Large amount | 0 (0.0) |  | 0 (0.0) |  |  |
| Pulmonary lesion location |  |  |  | 0.985 | 1.000 |
| No lesions | 0 (0.0) |  | 0 (0.0) |  |  |
| Upper left | 1 (0.9) |  | 0 (0.0) |  |  |
| Lower left | 1 (0.9) |  | 0 (0.0) |  |  |
| Upper right | 4 (3.7) |  | 1 (3.3) |  |  |
| Lower middle right | 0 (0.0) |  | 0 (0.0) |  |  |
| Multiple lobes or both lung | 101 (94.5) |  | 29 (96.7) |  |  |
| CRP (mg/L) | 11.42 (2.71, 36.07) |  | 76.22 ± 59.46 | -4.241 | **<0.001** |
| Hb (g/L) | 128.00 (111.00, 139.00) |  | 120.63 ± 17.88 | -1.143 | 0.253 |
| Platelet(×10^9^/L) | 269.00 (227.00, 322.00) |  | 316.56 ± 145.54 | -1.262 | 0.207 |
| Lymphocyte percentage (%) | 18.10 (13.40, 24.20) |  | 16.00 ± 7.24 | -2.004 | **0.045** |
| ALC(×10^9^/L) | 1.25 (0.97, 1.59) |  | 1.35 ± 0.52 | -0.023 | 0.981 |
| Monocyte percentage (%) | 7.10 (5.90, 9.00) |  | 7.35 ± 2.26 | -0.159 | 0.874 |
| AMC (×10^9^/L) | 0.51 (0.38, 0.70) |  | 0.68 ± 0.28 | -2.239 | **0.025** |
| Eosinophil percentage (%) | 1.60 (0.80, 3.00) |  | 1.05 (0.35, 2.25) | -2.193 | **0.028** |
| AEC (×10^9^/L) | 0.10 (0.05, 0.21) |  | 0.08 (0.04, 0.17) | -1.537 | 0.124 |
| Sputum smear grading |  |  |  | 18.890 | **<0.001** |
| No AFB | 9 (8.4) |  | 0 (0.0) |  |  |
| 1+ | 23 (21.5) |  | 2 (6.7) |  |  |
| 2+ | 17 (15.9) |  | 2 (6.7) |  |  |
| 3+ | 33 (30.8) |  | 7 (23.3) |  |  |
| 4+ | 25 (23.4) |  | 19 (63.3) |  |  |

Abbreviations: TB, tuberculosis; RR, rifampicin-resistant; MDR, multidrug-resistant; XDR, extensively drug-resistant; LZD, linezolid; BDQ, bedaquiline; DLM, delamanid; BMI, body mass index; HIV, human immunodeficiency virus; CRP, C-reactive protein; Hb, hemoglobin; ALC, absolute lymphocyte count; AMC, absolute monocyte count; AEC, absolute eosinophil count; AFB, acid-fast bacilli. SD, standard deviation; IQR, interquartile range; NA, not applicable.

**Table S7. Sociodemographic and Clinical Characteristics of MDR/RR-TB Patients with 6 Months of Treatment in External Cohort.**

| **Characteristics** | **Culture conversion (*n*=122)** |  | **No culture conversion (*n*=15)** | **χ^2^/z** | ***P*** |
| --- | --- | --- | --- | --- | --- |
|  | **n (%)/mean±SD/median (IQR)** |  | **n (%)/mean±SD/median (IQR)** |  |  |
| Age (years) | 41.5 (27.0, 57.0) |  | 51.80 ± 11.07 | -1.979 | **0.048** |
| Gender |  |  |  | 3.095 | 0.079 |
| Man | 78 (63.9) |  | 13 (86.7) |  |  |
| Woman | 44 (36.1) |  | 2 (13.3) |  |  |
| Occupation |  |  |  | 3.467 | 0.444 |
| Farmer | 0 (0.0) |  | 0 (0.0) |  |  |
| Worker | 18 (14.8) |  | 5 (33.3) |  |  |
| Service staff | 6 (4.9) |  | 0 (0.0) |  |  |
| Student | 7 (5.7) |  | 0 (0.0) |  |  |
| Unemployed individual | 29 (23.8) |  | 2 (13.3) |  |  |
| Others | 62 (50.8) |  | 8 (53.3) |  |  |
| Ethnicity |  |  |  | NA | NA |
| Han ethnicity | 122 (100.0) |  | 15 (100.0) |  |  |
| Non-Han ethnicity | 0 (0.0) |  | 0 (0.0) |  |  |
| Marital status |  |  |  | 3.719 | 0.170 |
| Single | 35 (28.7) |  | 1 (6.7) |  |  |
| Married | 85 (69.7) |  | 14 (93.3) |  |  |
| Divorced or widowed | 2 (1.6) |  | 0 (0.0) |  |  |
| Household registration |  |  |  | 2.349 | 0.125 |
| Rural | 58 (47.5) |  | 4 (26.7) |  |  |
| Urban | 64 (52.5) |  | 11 (73.3) |  |  |
| Current residence |  |  |  | 2.349 | 0.125 |
| Rural | 58 (47.5) |  | 4 (26.7) |  |  |
| Urban | 64 (52.5) |  | 11 (73.3) |  |  |
| BMI（kg/m^2^） | 19.49 (17.96, 20.91) |  | 18.26 ± 3.12 | -2.234 | **0.026** |
| Smoking habit |  |  |  | 0.642 | 0.423 |
| No | 78 (63.9) |  | 8 (53.3) |  |  |
| Yes | 44 (36.1) |  | 7 (46.7) |  |  |
| Quit smoking |  |  |  | 0.100 | 0.752 |
| No | 19 (15.6) |  | 2 (13.3) |  |  |
| Yes | 25 (20.5) |  | 5 (33.3) |  |  |
| Drinking habit |  |  |  | 0.184 | 0.668 |
| No | 107 (87.7) |  | 12 (80.0) |  |  |
| Yes | 15 (12.3) |  | 3 (20.0) |  |  |
| Quit drinking |  |  |  | NA | 0.559 |
| No | 9 (7.4) |  | 1 (6.7) |  |  |
| Yes | 6 (4.9) |  | 2 (13.3) |  |  |
| TB close contact |  |  |  | NA | 1.000 |
| No | 121 (99.2) |  | 15 (100.0) |  |  |
| Yes | 1 (0.8) |  | 0 (0.0) |  |  |
| Contact subjects |  |  |  | NA | NA |
| Relative | 1 (0.8) |  | 0 (0.0) |  |  |
| Friend | 0 (0.0) |  | 0 (0.0) |  |  |
| Colleague | 0 (0.0) |  | 0 (0.0) |  |  |
| TB treatment history |  |  |  | 3.899 | **0.048** |
| No | 48 (39.3) |  | 2 (13.3) |  |  |
| Yes | 74 (60.7) |  | 13 (86.7) |  |  |
| Duration of treatment history (years) | 0.3 (0.0,0.7) |  | 1.53 ± 1.30 | -3.586 | **<0.001** |
| Drug resistance types |  |  |  | 12.382 | **0.002** |
| RR | 18 (14.8) |  | 0 (0.0) |  |  |
| MDR | 69 (56.6) |  | 4 (26.7) |  |  |
| XDR | 35 (28.7) |  | 11 (73.3) |  |  |
| Number of resistant drugs | 2.0 (2.0,4.0) |  | 3.0 (2.0, 4.0) | -2.283 | **0.022** |
| Medication compliance |  |  |  | 29.908 | **<0.001** |
| No | 11 (9.0) |  | 10 (66.7) |  |  |
| Yes | 111 (91.0) |  | 5 (33.3) |  |  |
| Treatment regimens |  |  |  |  |  |
| Without LZD | 6 (4.9) |  | 6 (40.0) | 16.415 | **<0.001** |
| With LZD | 116 (95.1) |  | 9 (60.0) |  |  |
| Without BDQ | 90 (73.8) |  | 8 (53.3) | 1.828 | 0.176 |
| Witht BDQ | 32 (26.2) |  | 7 (46.7) |  |  |
| Without DLM | 116 (95.1) |  | 8 (53.3) | 22.465 | **<0.001** |
| With DLM | 6 (4.9) |  | 7 (46.7) |  |  |
| Comorbidity |  |  |  |  |  |
| Extrapulmonary TB |  |  |  | NA | 0.597 |
| No | 113 (92.6) |  | 15 (100.0) |  |  |
| Yes | 9 (7.4) |  | 0 (0.0) |  |  |
| Pneumoconiosis |  |  |  | NA | 1.000 |
| No | 117 (95.9) |  | 15 (100.0) |  |  |
| Yes | 5 (4.1) |  | 0 (0.0) |  |  |
| Malignant tumor |  |  |  | NA | 1.000 |
| No | 120 (98.4) |  | 15 (100.0) |  |  |
| Yes | 2 (1.6) |  | 0 (0.0) |  |  |
| Hepatitis |  |  |  | NA | 1.000 |
| No | 114 (93.4) |  | 14 (93.3) |  |  |
| Yes | 8 (6.6) |  | 1 (6.7) |  |  |
| Diabetes |  |  |  | 11.088 | **<0.001** |
| No | 89 (73.0) |  | 4 (26.7) |  |  |
| Yes | 33 (27.0) |  | 11 (73.3) |  |  |
| HIV |  |  |  | NA | NA |
| No | 122 (100.0) |  | 15 (100.0) |  |  |
| Yes | 0 (0.0) |  | 0 (0.0) |  |  |
| Rheumatism |  |  |  | NA | 1.000 |
| No | 121 (99.2) |  | 15 (100.0) |  |  |
| Yes | 1 (0.8) |  | 0 (0.0) |  |  |
| Cavity |  |  |  | 5.469 | **0.019** |
| No | 45 (36.9) |  | 1 (6.7) |  |  |
| Yes | 77 (63.1) |  | 14 (93.3) |  |  |
| Calcification |  |  |  | 2.218 | 0.136 |
| No | 110 (90.2) |  | 11 (73.3) |  |  |
| Yes | 12 (9.8) |  | 4 (26.7) |  |  |
| Mediastinal lymphadenopathy |  |  |  | 0.840 | 0.359 |
| No | 84 (68.9) |  | 8 (53.3) |  |  |
| Yes | 38 (31.1) |  | 7 (46.7) |  |  |
| Pleural effusion |  |  |  | NA | NA |
| No | 122 (100.0) |  | 15 (100.0) |  |  |
| Yes | 0 (0.0) |  | 0 (0.0) |  |  |
| Degree of pleural effusion |  |  |  | NA | NA |
| No effusion | 122 (100.0) |  | 15 (100.0) |  |  |
| Small amount | 0 (0.0) |  | 0 (0.0) |  |  |
| Moderate amount | 0 (0.0) |  | 0 (0.0) |  |  |
| Large amount | 0 (0.0) |  | 0 (0.0) |  |  |
| Pulmonary lesion location |  |  |  | 1.575 | 1.000 |
| No lesions | 0 (0.0) |  | 0 (0.0) |  |  |
| Upper left | 1 (0.8) |  | 0 (0.0) |  |  |
| Lower left | 1 (0.8) |  | 0 (0.0) |  |  |
| Upper right | 5 (4.1) |  | 0 (0.0) |  |  |
| Lower middle right | 0 (0.0) |  | 0 (0.0) |  |  |
| Multiple lobes or both lung | 115 (94.3) |  | 15 (100.0) |  |  |
| CRP (mg/L) | 12.99 (2.84, 49.18) |  | 80.55 ± 50.46 | -3.496 | **<0.001** |
| Hb (g/L) | 129.00 (113.25, 139.00) |  | 112.47 ± 16.50 | -2.737 | **0.006** |
| Platelet(×10^9^/L) | 269.00 (224.75, 322.50) |  | 350.53 ± 146.35 | -1.675 | 0.094 |
| Lymphocyte percentage (%) | 18.15 (12.83, 24.23) |  | 14.16 ± 6.07 | -2.185 | **0.029** |
| ALC(×10^9^/L) | 1.25 (0.97, 1.60) |  | 1.34 ± 0.55 | -0.155 | 0.877 |
| Monocyte percentage (%) | 7.20 (6.20, 9.00) |  | 6.20 ± 2.01 | -1.810 | 0.070 |
| AMC (×10^9^/L) | 0.53 (0.39, 0.72) |  | 0.65 ± 0.32 | -0.931 | 0.352 |
| Eosinophil percentage (%) | 1.60 (0.70, 3.10) |  | 0.85 ± 0.65 | -2.842 | **0.004** |
| AEC (×10^9^/L) | 0.10 (0.05, 0.21) |  | 0.08 ± 0.06 | -2.094 | **0.036** |
| Sputum smear grading |  |  |  | 9.313 | **0.033** |
| No AFB | 9 (7.4) |  | 0 (0.0) |  |  |
| 1+ | 25 (20.5) |  | 0 (0.0) |  |  |
| 2+ | 17 (13.9) |  | 2 (13.3) |  |  |
| 3+ | 37 (30.3) |  | 3 (20.0) |  |  |
| 4+ | 34 (27.9) |  | 10 (66.7) |  |  |

Abbreviations: TB, tuberculosis; RR, rifampicin-resistant; MDR, multidrug-resistant; XDR, extensively drug-resistant; LZD, linezolid; BDQ, bedaquiline; DLM, delamanid; BMI, body mass index; HIV, human immunodeficiency virus; CRP, C-reactive protein; Hb, hemoglobin; ALC, absolute lymphocyte count; AMC, absolute monocyte count; AEC, absolute eosinophil count; AFB, acid-fast bacilli. SD, standard deviation; IQR, interquartile range; NA, not applicable.

**Table S8. The relative importance ranking of predictor variables in models.**

| **Models** | **Logistic regression** | |  | **RF** | |  | **SVM** | |  | **GBDT** | |  | **EN** | |  | **ANN** | |
| --- | --- | --- | --- | --- | --- | --- | --- | --- | --- | --- | --- | --- | --- | --- | --- | --- | --- |
|  | **Relative importance  (%)** | **Ranking** |  | **Relative importance  (%)** | **Ranking** |  | **Relative importance  (%)** | **Ranking** |  | **Relative importance  (%)** | **Ranking** |  | **Relative importance  (%)** | **Ranking** |  | **Relative importance  (%)** | **Ranking** |
| **2 months of treatment** |  |  |  |  |  |  |  |  |  |  |  |  |  |  |  |  |  |
| Age | 24.81 | 2 |  | 43.49 | 1 |  | 21.46 | 2 |  | 44.73 | 1 |  | 28.39 | 2 |  | 2.58 | 7 |
| Drug resistance types | 10.39 | 4 |  | 4.83 | 5 |  | 7.43 | 4 |  | 2.02 | 7 |  | 10.94 | 4 |  | 2.21 | 10 |
| Medication compliance | 24.90 | 1 |  | 11.79 | 3 |  | 27.06 | 1 |  | 6.87 | 4 |  | 30.48 | 1 |  | 19.56 | 2 |
| Pneumoconiosis | 3.42 | 8 |  | 0.71 | 10 |  | 6.81 | 5 |  | 0.42 | 10 |  | 0.00 | 8 |  | 2.58 | 8 |
| Hepatitis | 1.72 | 9 |  | 1.22 | 9 |  | 6.25 | 7 |  | 0.65 | 9 |  | 0.00 | 9 |  | 13.65 | 4 |
| Diabetes | 10.37 | 5 |  | 3.32 | 7 |  | 17.03 | 3 |  | 4.47 | 5 |  | 9.76 | 5 |  | 2.58 | 9 |
| Cavity | 1.71 | 10 |  | 2.58 | 8 |  | 2.21 | 9 |  | 3.32 | 6 |  | 0.00 | 10 |  | 7.38 | 5 |
| Mediastinal lymphadenopathy | 4.93 | 7 |  | 3.71 | 6 |  | 1.97 | 10 |  | 0.91 | 8 |  | 0.77 | 7 |  | 26.20 | 1 |
| Lymphocyte percentage | 12.26 | 3 |  | 21.45 | 2 |  | 6.31 | 6 |  | 26.16 | 2 |  | 13.76 | 3 |  | 6.27 | 6 |
| Sputum smear grading | 5.49 | 6 |  | 6.91 | 4 |  | 3.46 | 8 |  | 10.46 | 3 |  | 5.91 | 6 |  | 16.97 | 3 |
| **6 months of treatment** |  |  |  |  |  |  |  |  |  |  |  |  |  |  |  |  |  |
| Age | 19.98 | 2 |  | 40.09 | 1 |  | 19.28 | 2 |  | 44.32 | 1 |  | 17.59 | 3 |  | 2.74 | 6 |
| TB treatment history | 14.49 | 4 |  | 5.78 | 6 |  | 12.28 | 4 |  | 4.19 | 5 |  | 15.72 | 4 |  | 47.26 | 1 |
| Treatment regimens with LZD | 18.05 | 3 |  | 6.48 | 4 |  | 14.80 | 3 |  | 7.48 | 4 |  | 19.54 | 2 |  | 14.38 | 3 |
| Treatment regimens with BDQ | 25.10 | 1 |  | 21.45 | 2 |  | 32.32 | 1 |  | 17.93 | 3 |  | 27.34 | 1 |  | 20.55 | 2 |
| Cavity | 9.91 | 6 |  | 5.89 | 5 |  | 10.32 | 6 |  | 3.20 | 6 |  | 9.51 | 6 |  | 11.64 | 4 |
| Sputum smear grading | 12.46 | 5 |  | 20.33 | 3 |  | 11.00 | 5 |  | 22.87 | 2 |  | 10.29 | 5 |  | 3.42 | 5 |

Abbreviations: RF, random forest; SVM, support vector machine; GBDT, gradient boosting decision tree; EN, elastic net; ANN, artificial neural network; LZD, linezolid; BDQ, bedaquiline.
